# The early COVID-19 pandemic and democratic attitudes

**DOI:** 10.1371/journal.pone.0253485

**Published:** 2021-06-22

**Authors:** Noam Lupu, Elizabeth J. Zechmeister

**Affiliations:** Department of Political Science, Vanderbilt University, Nashville, Tennessee, United States of America; Sogang University (South Korea), REPUBLIC OF KOREA

## Abstract

How does a public health crisis like a global pandemic affect political opinions in fragile democratic contexts? Research in political science suggests several possible public reactions to crisis, from retrospective anti-incumbency to rally ‘round the flag effects to democratic erosion and authoritarianism. Which of these obtains depends on the nature of the crisis. We examine whether and how the onset of the global pandemic shifted public opinion toward the president, elections, and democracy in Haiti. We embedded two experiments in a phone survey administered to a nationally representative sample of Haitians in April-June 2020. We find that the early pandemic boosted presidential approval and intentions to vote for the incumbent president, consistent with a rally effect. These results show that a rally effect occurs even in the most unlikely of places–an unstable context in which the incumbent president is struggling to maintain order and support. At the same time, we find scant evidence that the onset of the pandemic eroded democratic attitudes, even in a context in which democracy rests on uncertain grounds.

## Introduction

The COVID-19 pandemic arrived in the midst of a democratic recession. Globally, the public’s commitment to the most fundamental tenet of democracy–regular elections–has been wavering [1, 2]. Early research suggests that, much like the introduction of threats to national security from abroad, the COVID-19 pandemic may initially increase support for the incumbent administration [3, 4]. Some argue that such an inclination bodes well for democracy [5], while others warn that it includes a greater willingness to trade off basic freedoms [6, 7]. However, as is often the case, the majority of public opinion research related to the pandemic has focused on developed, wealthy democracies. How might a monumental health crisis shape attitudes in less developed contexts where the public’s commitment to the incumbent administration and democracy itself is already weaker?

We provide one answer to this question with data from survey experiments fielded in Haiti. Prior to the pandemic, Haitian politics was rife with corruption [8], waves of turbulent protests against an unpopular president had caused schools and businesses to close [9], support for democracy was low [10], and violence had become such a threat to public safety that even the annual Carnival celebration was canceled in February 2020 [11]. Given the weak position in which both the president and democracy found themselves at the dawn of the pandemic, Haiti was an unlikely case for a rally effect and a likely case for decreased support for democracy.

Our goal was to assess whether and how the mere appearance of a new crisis–the COVID-19 pandemic–would shift public opinion toward the president, elections, and democracy. To do so, we embedded two experiments in a phone survey that was administered to a nationally representative sample of Haitians in April-June 2020. The first experiment primed the pandemic: a random half received a 10-question COVID-19 module prior to a set of questions about presidential approval, commitment to elections, and democracy; the other half received these same questions after the political attitudes module. The second experiment randomly assigned individuals to consider the appropriateness of permitting the president to postpone elections in one of two conditions: violence or the pandemic.

The design unobtrusively primes the pandemic in order to observe what happens at the onset of a major public health crisis. Priming occurs when a factor (e.g., the pandemic) is made accessible and applicable to individuals’ judgments [12]. Priming effects work through one, or both, of the mind’s two core processing systems: uncontrolled/automatic or controlled/reasoned [13]. Our study was not designed to adjudicate between these mechanisms, yet we note that it was implemented when the public was not yet saturated with information about the pandemic. According to Johns Hopkins data from May 2, 2020, around the start of our survey, there were just under 100 confirmed cases of COVID-19 in Haiti. Further, only a limited set of social distancing policies was in place, and under-enforced [14]. In short, the public had little information to lean on when prompted to bring the pandemic to the “top of their heads”. Within this context, our study offers a unique perspective on how public opinion in a country beleaguered with institutional, economic, and social challenges bends when confronted with the specter of a major health crisis.

We find that priming the pandemic boosts presidential approval and intentions to vote for the incumbent president. These results show that a rally effect occurs even in the most unlikely of places–an unstable context in which the incumbent president is struggling to maintain order and support. We also show that this inclination to rally is accompanied by a willingness to cede additional power to the president: the pandemic increases the public’s willingness to tolerate the president unilaterally delaying elections. At the same time, the results demonstrate that–even in a context in which democracy rests on uncertain grounds–a decline in fundamental support for democracy is not inherent in the pandemic.

To be clear, support for democracy does not rest on firm foundations in Haiti. But whether core democratic values slip further depends not on the mere appearance of a new crisis, but rather on the politics that will follow.

### Crises, disasters, and democratic attitudes

How does the mass public respond to negative events like the pandemic? According to existing scholarship, the answer may depend on the nature of the event. One line of scholarship, building on the vast body of research on economic voting [15], argues that the public holds the executive responsible for poor outcomes. The *blind retrospection* model, as Achen and Bartels label it, holds that individuals lash out against political leaders when bad things happen [16]. Events theorized to produce a turn against the executive include shark attacks, droughts, shifts in the global economy, and defeats in college sports [16–20].

Some argue that negative outcomes that are very clearly outside the executive’s control undermine incumbent support, in some cases even when the exogenous nature of the shock is made explicit [19, 21]. Ashworth, Bueno de Mesquita, and Friedenberg highlight that it may be rational for voters to blame incumbents for how they respond to a crisis even if they cannot be blamed for causing it [22]. Others argue that the tendency to punish incumbents is more likely to emerge when the public is assisted in making a connection between the negative event and political leadership. For example, the shark attacks that sank President Wilson’s popularity in 1916 were beyond the administration’s control, yet they were politicized by their proximity to an election, media discussion, political cartoons, the presence of federal officials following incidents, and the toll the attacks took on the local economy [16]. If the public’s response to the onset of a pandemic is consistent with blind retrospection, we would expect a decrease in support for the president, either because of an impulsive tendency to punish leaders for poor outcomes or because the public has been supplied a frame that connects the negative event to the president’s (poor) leadership.

Another possibility is that the pandemic represents the type of national crisis that research suggests causes individuals to *rally ‘round the flag*, increasing their support for the incumbent and, in some cases, related attitudes [23, 24]. Mueller theorized that certain events–those that are jointly sudden and intense, have an international component, and are relevant to the executive office–cause the public to unite in support of incumbent leadership [23]. Though the rally effect may dissipate as media attention and consensus-promoting elite rhetoric fades, the initial response is presumed to be a psychological shift toward in-group consolidation in reaction to a collective threat [25]. Since the executive office is symbolic of the nation, the incumbent benefits from increased support. At the same time, individuals may also increase their feelings of patriotism and support for other core symbols and institutions [26–28]. If the pandemic generated a rally, we should see the opposite of blind retrospection: increased support for the incumbent executive.

These two frameworks are silent on the consequences of opinion shifts for a broader set of democratic attitudes. For broader perspective, then, we turn to political psychology research that suggests that individuals cope with major, lethal threats by increasing their deference to leadership and expressions of authoritarian attitudes. From one perspective, increasing evaluations of incumbent leadership is a way to combat distress, as the leader becomes a proxy for efficacy and power [29, 30]. From another perspective, conditions that make mortality salient provoke an ego-defensive reaction that includes shifting support for incumbent leadership and authoritarian attitudes [31, 32]. In either case, the prediction is similar: collective crises provoke a turn in the public that has two core components. The first is consistent with a rally effect: increased support for incumbent leaders. The second is a decline in adherence to basic democratic principles. Scholars have found that events as varied as economic decline, political threat, security threats, and natural disasters can trigger such authoritarian turns [30, 33–35]. Should the onset of the COVID-19 pandemic be added to this list?

One way to uncover how the public responds to a global pandemic is to assess the extent to which public opinion dynamics comport with each of these theoretical frameworks. If the retrospection model obtains, we should see the pandemic reduce incumbent support. If the pandemic generates a rally effect, however, we should see the opposite: increased incumbent support. Finally, if the threat/authoritarian model prevails, we ought to find *both* increased incumbent support and erosion in core democratic values. To be clear, both the rally and threat/authoritarian frameworks predict an increase in incumbent support. Yet the threat/authoritarian model goes a step further, implicating democratic attitudes.

Which of these schools of thought is likely to capture *early* public opinion dynamics in a country confronting the COVID-19 pandemic? In theory, this is an open question. The onset of a pandemic is clearly a negative shock to the public’s welfare, a precondition for blind retrospection. To be sure, a quick, bold, and effective response could mitigate against such an outcome. Yet, this was not the case in Haiti, where initial measures were weak [14] and the public overwhelmingly critiqued that effort (see mean responses to COVID3 in Table 1 in S1 File). At the same time, the pandemic has an international element, as it originated outside the country (everywhere but China), a precondition for a rally-inducing event. And the pandemic introduces the specter of substantial threat and uncertainty that the threat/authoritarian model identifies as central to outcomes.

Some empirical work has found that the onset of the pandemic resulted in increased support for incumbent leadership [3, 4], which could be consistent with both the rally and the threat/authoritarian models. While some have come to rosy conclusions regarding public opinion dynamics and democracy [5], others have identified opinion shifts that could be more in line with an authoritarian turn, such as greater willingness to trade off basic freedoms [6, 7]. With theory and empirical evidence inconclusive, we turn to an experimental design that reveals public opinion dynamics in response to priming the pandemic in its early stage.

### Experimental design

Our data come from a national cellphone survey we fielded in Haiti from April 23 to June 10, 2020 with 2,028 adult respondents. An experienced local survey firm drew the sample and recruited enumerators. All the interviews were recorded and audited for quality control by both the local firm and our research team. Sampling relied on random digit dialing supplemented by frequency matching to ensure balance in terms of region, gender, and age cohorts. According to AmericasBarometer data from 2017, 87% of Haitian households have a cellphone; we calculated post-stratification weights to approximate the population, though we also assess the experiment without weights in the appendix [36]. Details on the sampling methodology and weighting are in Appendix 1 in S1 File.

We obtained voluntary and informed consent from participants using an IRB-approved consent protocol. Our research was overseen by the Institutional Review Board at Vanderbilt University. We did not use deception. Since the survey was conducted over the phone, informed consent was obtained verbally from all respondents prior to beginning the survey. The consent script was programmed into the computer software used by interviewers; they had to click that the respondent had consented in order to proceed to the questionnaire. Respondents also consented to have the interview recorded for quality control purposes. Haiti does not require permits for foreign research of this type.

The questionnaire was structured such that half the respondents were assigned to a COVID-19 prime condition in which they were asked a module of ten questions about their views on the pandemic and then a module on various topics that included the dependent variables of interest: presidential approval, support for postponing elections, tolerance for coups, and support for democracy in the abstract. The other half–the control condition–answered these other questions first, and then the ten-question pandemic module. We analyze only the subset of the questionnaire that pertains to this study; the full instrument, designed to capture data on various issues related to democratic governance, is documented in Appendixes 2, 3 and 5, 6 in S1 File. The goal of the COVID-19 prime condition was to raise the accessibility and availability of the pandemic in people’s judgments, compared to the control group. Random assignment resulted in homogenous groups on observable sociodemographic variables (see Table 3 in S1 File). In addition, within the survey, one question–about a hypothetical decision by the president to postpone elections–was programmed to ask a random half of the respondents whether this action is justifiable in the case of a public health emergency, like the pandemic, while another random half was asked about the case of violence. We elected to use these nonobtrusive means to assess the public opinion consequences of the crisis in order to reduce the potential for demand effects [37]. Given the design and timing of the study, the data permit insight into how public opinion shifts in the early stages of the pandemic.

## Results: Attitudes toward the president

Table 1 shows simple difference of means test results for the weighted sample, for a set of measures related to support for the president and deference to his authority. In Tables 4–7 in S1 File, we assess the robustness of these results to using the unweighted data and to including demographic control variables. With respect to presidential approval, the survey included a standard question that asks respondents how they would rate the job performance of Haitian President Jovenel Moïse. As expected, mean levels of approval are low: 2.11 on a 1–5 scale. Still, those in the primed condition report significantly higher levels of presidential approval (2.16) compared to the level of presidential approval expressed in the control condition (2.00). In addition, the survey asked about future vote intention (if an election were held today). The overwhelming majority report that they would not vote or would nullify their ballot, and only 7% report that they would vote for the incumbent. Yet, we see some differences across the prime and control conditions–but note that these differences are not very robust (see S1 File). In brief, despite a near complete deficit of support for the executive, the pandemic nudges individuals mildly in the direction of a rally, consistent with findings in other contexts [3, 4].

**Table 1:** Prime experiment results: Attitudes toward the president.

	Prime	Control	Treatment Effect
Approval (1–5)	2.16	2.00	**0.17**
Intention to vote for president (%)	8.18	5.15	**3.03**
Postpone elections in health crisis (%)	88.90	79.72	**9.17**
Postpone elections in high violence (%)	77.17	77.32	-0.15

*Notes*: Bolded differences are statistically significant at 95% (two-tailed).

While rally ‘round the flag scholarship focuses mainly on increases in support for the executive, the threat/authoritarian framework suggests such turns are accompanied by increased deference to authority. To assess that expectation, we ask to what extent the specter of crisis increases individuals’ willingness to accept a unilateral move by the president to postpone elections. We assigned a random half of the respondents to consider whether it can be justifiable for the president of the country to postpone elections under one of two conditions: “a public health emergency like the coronavirus” or “when there is a lot of violence.” A higher proportion of respondents indicated that postponing elections is acceptable when violence is high (89%) than in a public health emergency like coronavirus (80%); the difference of proportions is significant at *p = 0*.*002*.

In addition, we consider whether expressed tolerance for the president postponing elections is higher among those who were made to reflect on the pandemic via pre-test exposure to the 10 question COVID-19 module. Those in the primed condition are 9.2 percentage points more likely to support permitting the president the authority to postpone elections in a public health emergency. In contrast, the results reveal no difference across conditions for those asked about a lot of violence. In short, the prime uniquely shapes attitudes on the politics of the pandemic, such that individuals increase their support for the president and their deference to his authority to postpone elections.

## Results: Support for democracy and coups

To what extent does the crisis bode poorly for a broader set of attitudes related to democracy? To gauge this, we first consider responses to the classic support for democracy question, which asks respondents their level of agreement with the notion that, despite its problems, democracy is better than any other form of government. As expected, the data reveal a significant amount of democratic ambivalence in Haiti: the mean response on the 1–7 response scale is 4.00. Yet, there is no statistically significant difference between the mean values on this indicator between those who were primed with the COVID-19 module and those who were not (see Table 2). Priming the pandemic seems to have no corrosive effect on support for democracy. It is worth noting that this result also surfaces in a study that considers the effect of the disastrous magnitude 7.0 earthquake on public support for democracy in Haiti in 2010: levels did not change to a significant degree after the event (see the AmericasBarometer data on Haiti, available at www.vanderbilt.edu/lapop) [38].

**Table 2:** Prime experiment results: Attitudes toward democracy and coups.

	Prime	Control	Treatment Effect
Support for democracy (1–7)	4.02	3.99	0.03
Coup is justified in high crime (%)	46.34	47.62	-1.27
Coup is justified in high corruption (%)	39.57	46.54	-6.97
Coup is justified in health crisis (%)	41.68	40.94	0.74

*Notes*: No differences are statistically significant at 95% (two-tailed).

Next, we consider means on a set of questions that ask about tolerance for coups under certain conditions: “a lot of crime”, “a lot of corruption”, or “a public health emergency like the coronavirus.” Haiti does not have a military, but it does have a national police (the Police Nationale d’Haïti); therefore, the coups reference the notion of this force taking power. Respondents were randomly assigned to one of the first two conditions, and then all responded to the latter condition. Generally speaking, tolerance for coups under poor conditions is high in Haiti: the mean proportion finding a coup justifiable across these questions is 43.0. Yet, at the same time, the proportion of respondents who assert a coup is justifiable under conditions of public health emergency is, in fact, lower than this average, at 41.7%. Importantly, none of the values on these indicators of support for coups–whether under conditions of corruption, crime, or public health emergency–is significantly higher in the COVID prime, versus the control, condition. Unlike other anxiety-inducing collective threats in other contexts, the onset of the pandemic does not appear to have bolstered a broader set of authoritarian attitudes in Haiti.

## Conclusion

How does a public health crisis like a global pandemic affect political opinions in fragile democratic contexts? Research in political science suggests several possible public reactions to crisis, but which of these obtains in a global pandemic depends on how the public perceives the crisis. Does the public lash out against the incumbent in a manner consistent with the blind retrospection model, does it rally around the executive as if the pandemic were an act of war, or does it shift in deference to authority *and* authoritarian principles? Our survey experiments in Haiti reveal that public opinion at the onset of the COVID-19 pandemic moved in a manner consistent with the kinds of rally effects documented in contexts of interstate conflict. By uncovering similar rally effects in a fragile and developing democracy like Haiti, this study bolsters the generality of this theoretical model, not only to a health crisis but also to a distinct context.

We further find some evidence of increased deference toward the executive’s authority. This may be an under-explored outgrowth of rally dynamics. Indeed, then-Mayor of New York City Rudy Giuliani floated postponing his departure from office while riding a wave of approval following the 9/11 terrorist attacks [39, 40]. Increased deference to the executive is also consistent with the threat/authoritarian model. However, we find no evidence of a broader shift in democratic attitudes. Our results are reassuring for those who worry that the pandemic will inevitably undermine democratic values.

Globally, the pandemic was already well underway by the time it reached Haiti and we fielded our survey. This meant that COVID-19 was already known to some Haitians, a good deal of whom were already taking precautions to avoid infection. An experiment like ours requires that the issue being primed is not already so saturated that the treatment has no discernible effect. Since we fielded our survey early in the pandemic in Haiti, we were able to leverage the fact that there was still some variation in the extent to which individuals were pretreated with relevant information. Still, the fact that it was already on the minds of many of our respondents may be why our prime only had substantively small effects. It may likewise be the case that shifts in support for the president are modest when a crisis confronts an executive with particularly low approval, as was the case in Haiti.

It is important to be careful about extrapolating from our study to the COVID-19 pandemic as a whole. We fielded our survey during the very early stages of the pandemic in Haiti, allowing us to document its early effects on public opinion. But the global pandemic is still ongoing, and both its development and the Haitian government’s response to it may well affect political opinions as well. In fact, the nature of the crisis could move toward one more consistent with the retrospective evaluation model if frames emerge that suggest the government’s response is deficient. Still, our study documents the effects of the onset of the pandemic on important political attitudes, even if those effects change as the pandemic itself progresses.

## Supporting information

S1 File Supporting information. (DOCX)
