# Supplementary material for: The early COVID-19 pandemic and democratic attitudes
Source: PLoS One. 2021 Jun 22;16(6):e0253485. doi: 10.1371/journal.pone.0253485 (PMC8219124; doi:10.1371/journal.pone.0253485)
Supplement: S1 File — (DOCX) [file pone.0253485.s001.docx]

**Supporting Information**

**“The early COVID-19 pandemic and democratic attitudes”**

Noam Lupu and Elizabeth J. Zechmeister

**Table of Contents**

1. Sampling Methodology and Weights 2
2. Pandemic Question Module 3

Table 1. Pandemic Module Treatment Effects 4

1. Political Attitudes Module 5

Table 2. Political Attitudes Module Treatment Effects 8

1. Auxiliary Analyses 9

Table 3. Balance Test 9

Table 4. Attitudes toward the President, Without Weights 10

Table 5. Attitudes toward Democracy and Coups, Without Weights 10

Table 6. Attitudes toward the President, With Controls 11

Table 7. Attitudes toward Democracy and Coups, With Controls 12

1. Complete survey questionnaire (English) 13
2. Complete survey questionnaire (Haitian Creole) 41

**1. Sampling Methodology and Weights**

The firm, d.a.g.m.a.r. Research, has a mobile phone database of 200,000 numbers split evenly across each of Haiti’s 10 departments: Artibonite, Centre, Grand’Anse, Nippes, Nord, Nord-Est, Nord-Ouest, Ouest, Sud, Sud-Est.

The sample was stratified by region (North, Center, West, and South) with targets set proportional to population, and additional targets were set by age and gender to match census projections. Only individuals 18 or older were eligible. Phone numbers were called twice before being discarded.

Weights were created by raking over indicators for age group (census projections provided by the survey firm), gender (50-50 split), education, and a module on ownership of household items. Since census data in Haiti are outdated (the last census was in 2003) and only partially public, we used the 2017 AmericasBarometer survey of Haiti as the population benchmark for education and ownership of household items. Missing values in the current survey were imputed using chained imputation to ensure each observation received a weight.

**2. Pandemic Question Module** **[English translation]**

**Survey Code, Question Wording, and Variable Coding:**

1. COVID1. How serious of a problem do you think the coronavirus outbreak is for Haiti? (coded 1-5, greater values = more serious)
2. COVID2A. How worried are you about the possibility that you or someone in your household will become sick with the coronavirus? (coded 1-4, greater values = more worried)
3. COVID2B. How worried are you about the possibility that your household economic situation will be negatively affected by the coronavirus? (coded 1-4, greater values = more worried)
4. COVID2C. How worried are you about the possibility that your household will have difficulty accessing basic goods, such as food or medicine, due to the coronavirus? (coded 1-4, greater values = more worried)
5. COVID3. How would you rate the performance of the national government in handling the coronavirus outbreak? (coded 1-5, higher values = better evaluations)
6. COVID4. Coronavirus is spreading in Haiti. Who is the most responsible for the increased number of infections? (coded 1=incumbent administration, 0=other)
7. COVID6. Please tell me if you have taken any of the following actions because of the recent coronavirus outbreak. Have you canceled any plans to attend large gatherings such as social events or church? (1=yes)
8. COVID7. Have you kept a greater distance between you and others when out in public? (1=yes)
9. COVID8. Have you washed your hands more often with water and soap or sanitizer? (1=yes)
10. COVID14. Considering those who get the coronavirus, would you say that they deserve to be sick because of the way they live, or that they could not help becoming sick? (1=deserve to be sick)

**Table 1**. *Pandemic Module Treatment Effects*

| **Variable** | **Prime (N=1,002)** | **Control (N=1,026)** | **Difference** |
| --- | --- | --- | --- |
| COVID1 | 4.13 | 4.36 | **-0.23** |
| Covid2a | 3.07 | 3.12 | -0.06 |
| Covid2b | 3.07 | 3.14 | -0.06 |
| Covid2c | 2.88 | 2.83 | 0.05 |
| Covid3 | 2.05 | 2.18 | -0.13 |
| Covid4 | 0.55 | 0.55 | 0.00 |
| Covid6 | 0.73 | 0.76 | -0.03 |
| Covid7 | 0.90 | 0.88 | 0.02 |
| Covid8 | 0.97 | 0.98 | -0.01 |
| Covid14 | 0.12 | 0.10 | 0.02 |

*Note:* Values are coded as noted above and survey weights are applied. Bolded differences are statistically significant at 95% (two-tailed).

**3. Political Attitudes Module** **[English translation]**

The survey included 25 other attitudinal questions within the core questionnaire (below), outside the scope of our study; in the interest of transparency, we assessed the treatment effects on each of these variables (see Table S2 below). We find significant results on four of these. Significant results on these four indicate that there are additional positive spillover effects in the inclination toward more favorable evaluations of the national economy, the quality of drinkable water in the country, the state of the electoral process in Haiti, and the extent to which politicians in Haiti are (not) corrupt. In all cases, evaluations are negative on average, but marginally higher (more favorable) in the primed condition. Jointly, these results provide additional support for a conclusion that priming the pandemic leads to a rally effect.

**Survey Code, Question Wording, and Variable Coding** (study variables in italics):

SOCT2. Do you think that the country’s current economic situation is better than, the same as or worse than it was 12 months ago? (coded 1-3, higher values = better)

IDIO2. Do you think that your economic situation is better than, the same as, or worse than it was 12 months ago? (coded 1-3, higher values = better)

In general, how would you rate the quality of each of the following services in Haiti?

HAIACS5. Drinkable water. (coded 1-5, higher values = better)

HAIACS6. Trash disposal. (coded 1-5, higher values = better)

ANESGI1. Do you think what happens generally to people in your neighborhood [split sample: Haitians in this country] will have something to do with what happens in your life? (1=yes)

IT1N1. And speaking of the people from your neighborhood [split sample: Haitians], would you say that people in your community are very trustworthy, somewhat trustworthy, not very trustworthy or untrustworthy...? (coded 1-4, higher values = more trustworthy)

*Now, changing the subject. Some people say that under some circumstances it would be justified for the Police Nationale d’Haïti to take power by a coup d’état (military coup). In your opinion would a coup by the police be justified…:*

*JC10. When there is a lot of crime. (1=justified; asked only of split sample)*

*JC13. When there is a lot of corruption. (1=justified; asked only of split sample)*

*JC13COVID. When there is a public health emergency like the coronavirus (1=justified)*

*JCCOV1. Do you believe that when there is a public health emergency like the coronavirus it is justifiable for the president of the country to postpone elections? (1=justified; asked only of split sample)*

*JCCOV2. Do you believe that when there is a lot of violence it is justifiable for the president of the country to postpone elections? (1=justified; asked only of split sample)*

VIC1EXT. Now, changing the subject, have you been a victim of any type of crime in the past 12 months? That is, have you been a victim of robbery, burglary, assault, fraud, blackmail, extortion, violent threats or any other type of crime in the past 12 months? (1=yes)

AOJ11. Speaking of the neighborhood where you live and thinking of the possibility of being assaulted or robbed, do you feel very safe, somewhat safe, somewhat unsafe or very unsafe? (coded 1-4, with higher values=more safe)

IVOL24. Is there a criminal gang or gangs in your neighborhood? (yes=1; those who responded “1” answered 3 follow-up questions)

B1. To what extent do you think the courts in Haiti guarantee a fair trial? (coded 1=7, higher values = more)

B2. To what extent do you respect the political institutions of Haiti? (coded 1=7, higher values = more)

B3. To what extent do you think that citizens’ basic rights are well protected by the political system of Haiti? (coded 1=7, higher values = more)

B4. To what extent do you feel proud of living under the political system of Haiti? (coded 1=7, higher values = more)

B6. To what extent do you think that one should support the political system of Haiti? (coded 1=7, higher values = more)

BESS1. To what extent do you think most people would try to take advantage of you if they got the chance? (coded 1=7, higher values = more)

M1. Speaking in general of the current administration, how would you rate the job performance of President Jovenel Moïse? (coded 1-5, higher values=better)

HAIM6. Would you say that the election process in Haiti is very good, good, neither good nor bad, bad, or very bad? (coded 1-5, higher values=better)

*ING4. Democracy may have problems, but it is better than any other form of government. To what extent do you agree or disagree with this statement? (coded 1-7, higher values=more agreement)*

EXC7. Taking into account your own experience or what you have heard, corruption among public officials is: (coded 1-5, higher values=less common; asked of split sample)

EXC7NEW. Thinking of the politicians of Haiti… how many of them do you believe are involved in corruption? (coded 1-5, higher values=less common; asked of split sample)

ESSH1. How is your health in general? (coded 1-5, higher values=better)

HC3. Over the last 12 months that is from April 2019/May 2019 until today have you or any household member needed medical attention? (yes=1; those who responded “1” answered 3 follow-up questions)

SD6NEW2H. And thinking of the quality of public medical and health services in general? Are you very satisfied, satisfied, dissatisfied, or very dissatisfied? (coded 1-4, higher values=more satisfied)

POL1A. How much interest do you have in politics: a lot, some, little or none? (coded 1-4, higher values = more)

*VB20A. If the next presidential elections were being held this week, what would you do? (coded so that 1= vote for the incumbent, 0 otherwise)*

HAIRIG1. Do you think that now you know more, about the same, or less about your rights than 12 months ago? (coded 1-3, higher values=know more; asked of a split sample)

HAIRIG5. Do you feel you know more, about the same, or less about your rights to access to justice than 12 months ago? (coded 1-3, higher values=know more; asked of a split sample)

WF1. Do you or someone in your household receive regular assistance in the form of money, food, or products from the government, not including pensions/social security? (1=yes)

**Table 2.** *Political Attitudes Module Treatment* *Effects*

| **Variable** | **Prime (N=1,002)** | **Control (N=1,026)** | **Difference** |
| --- | --- | --- | --- |
| Soct2 | 1.04 | 1.02 | **0.03** |
| Idio2 | 1.06 | 1.06 | 0.00 |
| Haiacs5 | 2.10 | 1.93 | **0.18** |
| Haiacs6 | 2.19 | 2.05 | 0.14 |
| Anesgi | 0.72 | 0.76 | -0.04 |
| It1n | 2.07 | 2.11 | -0.05 |
| Vic1ext | 0.23 | 0.23 | -0.00 |
| Aoj11 | 2.29 | 2.34 | -0.05 |
| Ivol24 | 0.21 | 0.22 | -0.01 |
| B1 | 2.32 | 2.36 | -0.03 |
| B2 | 4.12 | 3.97 | 0.15 |
| B3 | 2.42 | 2.34 | 0.08 |
| B4 | 2.82 | 2.66 | 0.16 |
| B6 | 3.35 | 3.22 | 0.13 |
| Bess1 | 4.09 | 4.21 | -0.12 |
| Haim6 | 2.10 | 1.91 | **0.19** |
| Exc7 | 2.96 | 2.93 | 0.04 |
| Exc7new | 2.33 | 2.12 | **0.22** |
| Essh1 | 2.81 | 2.78 | 0.03 |
| Hc3 | 0.68 | 0.69 | -0.00 |
| Sd6new2h | 2.13 | 2.11 | 0.02 |
| Pol1 | 1.97 | 2.08 | -0.11 |
| Hairig1 | 1.94 | 2.05 | -0.11 |
| Hairig5 | 1.85 | 1.87 | -0.03 |
| Wf1 | 0.01 | 0.01 | 0.00 |

*Note:* Values are coded as noted above and survey weights are applied. Bolded differences are statistically significant at 95% (two-tailed).

**4. Auxiliary Analyses**

**Table 3**. *Balance Test* *for COVID-19 Prime Experiment*

| **Variable** | | **(1)** |
| --- | --- | --- |
| Female | | -0.004 |
|  |  | (0.023) |
| Age group | |  |
|  | 18-25 | -- |
|  |  | -- |
|  | 26-35 | 0.022 |
|  |  | (0.032) |
|  | 36-45 | 0.007 |
|  |  | (0.035) |
|  | 46-55 | -0.018 |
|  |  | (0.036) |
|  | 56-65 | 0.015 |
|  |  | (0.053) |
|  | 66+ | 0.075 |
|  |  | (0.081) |
|  |  |  |
| Education | |  |
|  | Incomplete primary | -- |
|  |  | -- |
|  | Primary | -0.042 |
|  |  | (0.077) |
|  | Secondary | -0.061 |
|  |  | (0.072) |
|  | Post-Secondary | -0.061 |
|  |  | (0.075) |
|  |  |  |
| Region | |  |
|  | North | -- |
|  |  | -- |
|  | Center | 0.026 |
|  |  | (0.035) |
|  | West | 0.014 |
|  |  | (0.031) |
|  | South | -0.001 |
|  |  | (0.035) |
|  |  |  |
| Constant | | 1.545^*^ |
|  | | (0.078) |
| Observations | | 1,983 |
| *R^2^* | | 0.0024 |

OLS regression; standard errors in parentheses; ^*^ p<0.05

**Table 4**. *Attitudes toward the President, Without Weights*

|  | **Prime** | **Control** | **Treatment Effect** |
| --- | --- | --- | --- |
| Approval (1-5) | 2.18 | 2.03 | **0.15** |
| Intention to vote for president (0-1) | 6.94 | 6.15 | 0.79 |
| Postpone elections in health crisis (%) | 88.18 | 81.65 | **6.53** |
| Postpone elections in high violence (%) | 76.28 | 79.04 | -2.76 |

*Notes*: Bolded differences are statistically significant at 95% (two-tailed).

**Table 5**. *Attitudes toward Democracy and Coups, Without Weights*

|  | **Prime** | **Control** | **Treatment Effect** |
| --- | --- | --- | --- |
| Support for democracy (1-7) | 4.07 | 3.98 | 0.09 |
| Coup is justified in high crime (%) | 42.73 | 43.50 | -0.77 |
| Coup is justified in high corruption (%) | 37.43 | 41.77 | -4.34 |
| Coup is justified in health crisis (%) | 39.53 | 36.94 | 2.59 |

*Notes*: No differences are statistically significant at 95% (two-tailed).

**Table 6**. *Attitudes toward the President, With Controls*

| **Variable** | **Presidential Approval** | **Presidential Vote Intention** | **Postpone Elections due to Health Crisis** | **Postpone Elections due to Violence** |
| --- | --- | --- | --- | --- |
| Prime | -0.173^*^ | -0.470 | -0.720^*^ | -0.104 |
|  | (0.068) | (0.264) | (0.248) | (0.213) |
| COVID concern | 0.027 | 0.157 | 0.007 | 0.116 |
|  | (0.029) | (0.104) | (0.100) | (0.092) |
| Female | -0.095 | -0.880^*^ | 0.159 | 0.180 |
|  | (0.068) | (0.262) | (0.247) | (0.218) |
| Age | -0.003 | -1.08 | -0.149 | -0.058 |
|  | (0.028) | (0.086) | (0.100) | (0.082) |
| Education | 0.143^*^ | -0.303 | 0.331 | 0.079 |
|  | (0.062) | (0.193) | (0.206) | (0.184) |
|  |  |  |  |  |
| Constant | 2.237^*^ | -1.185 | 1.610^*^ | 0.549 |
|  | (0.212) | (0.689) | (0.792) | (0.627) |
| Region dummies | ✓ | ✓ | ✓ | ✓ |
| Observations | 1,463 | 1,384 | 771 | 728 |
| *R^2^* | 0.048 | – | – | – |

OLS regression, first data column, and logit regression for others; standard errors in parentheses; ^*^ *p<0.05*

**Table 7**. *Attitudes toward Democracy and Coups, With Controls*

| **Variable** | **Support for Democracy** | **Coup Justified in High Crime** | **Coup Justified in High Corruption** | **Coup Justified in Health Crisis** |
| --- | --- | --- | --- | --- |
| Prime | 0.103 | 0.008 | 0.315 | 0.010 |
|  | (0.146) | (0.209) | (0.215) | (0.146) |
| COVID concern | -0.074 | -0.044 | -0.112 | 0.033 |
|  | (0.068) | (0.094) | (0.097) | (0.066) |
| Female | -0.189 | 0.475^*^ | 0.489^*^ | 0.451^*^ |
|  | (0.147) | (0.215) | (0.209) | (0.147) |
| Age | -0.011 | -0.390^*^ | -0.260^*^ | -0.322^*^ |
|  | (0.060) | (0.094) | (0.093) | (0.064) |
| Education | 0.117 | -0.778^*^ | -0.305 | -0.771^*^ |
|  | (0.138) | (0.214) | (0.207) | (0.137) |
|  |  |  |  |  |
| Constant | 4.271^*^ | 2.442^*^ | 1.241 | 1.654^*^ |
|  | (0.459) | (0.712) | (0.646) | (0.459) |
| Region dummies | ✓ | ✓ | ✓ | ✓ |
| Observations | 1,561 | 602 | 604 | 1,269 |
| *R^2^* | 0.007 | – | – | – |

OLS regression, first data column, and logit regression for others; standard errors in parentheses; ^*^ *p<0.05*

**5. Complete survey questionnaire (English)**

*AmericasBarometer 2018/19 Haiti Questionnaire Version # 12.0.10.0 -* ***IRB Approval #:*** *170509*

| ****** | | |
| --- | --- | --- |
| ****** |  | ****** |

**LAPOP: Haiti, 2020**

© Vanderbilt University 2020. All rights reserved.

| **PAIS. Country**:   \| 01. Mexico \| 02. Guatemala \| 03. El Salvador \| 04. Honduras \| 05. Nicaragua \| \| --- \| --- \| --- \| --- \| --- \| \| 06. Costa Rica \| 07. Panama \| 08. Colombia \| 09. Ecuador \| 10. Bolivia \| \| 11. Peru \| 12. Paraguay \| 13. Chile \| 14. Uruguay \| 15. Brazil \| \| 16. Venezuela \| 17. Argentina \| 21. Dom. Rep. \| **22. Haiti** \| 23. Jamaica \| \| 24. Guyana \| 25. Trinidad & Tobago \| 26. Belize \| 40. United States \| 41. Canada \| \| 27. Suriname \| 28. Bahamas \| 29. Barbados \| 30. Grenada \| 31. Saint Lucia \| \| 32. Dominica \| 33**.** Antigua and Barbuda \| 34. Saint Vincent and the Grenadines \| 35. Saint Kitts and Nevis \|  \| |  |
| --- | --- | --- | --- | --- | --- | --- | --- | --- | --- | --- | --- | --- | --- | --- | --- | --- | --- | --- | --- | --- | --- | --- | --- | --- | --- | --- | --- | --- | --- | --- | --- | --- | --- | --- | --- | --- |
| **IDNUM.** Questionnaire number **[assigned at the office]** |  |
| **Q2.** How old are you? ________ years **[RECORD AGE IN YEARS COMPLETED. Age cannot be less than 18 years]** |  |
| **CIUDADANO1T.** To begin, are you a Haitian citizen or a permanent resident of Haiti? (1) Yes (2) No |  |
| **Q1T.** Could you tell me your gender? (1) Male (2) Female (3) Other |  |
| **PROV1T.** What department do you live in? __________________ |  |
| **MUNICIPIO1T.** Which commune in the department do you live in? _____________ |  |
| **UR1.** Do you live in an urban area of the commune, or in a rural area of the commune?  (1) Urban area (2) Rural area  (888888) **[DON’T READ]** Don’t know (988888) **[DON’T READ]** No answer |  |
| **REGION:**  (1) North  (2) Center  (3) West  (4) South |  |
| **IDIOMAQ. Questionnaire language:** (14) Kreyòl |  |
| **Start time:** _____:_____ |  |
| **FECHA. Date Day**: ____ **Month:_______**  Year: 2020 |  |
| **ATTENTION: It is compulsory to always read the STUDY INFORMATION SHEET and obtain consent before starting the interview.** | |

| **QUESTIONNAIRE A**  To begin, we would like to ask you some questions about the coronavirus situation. | |
| --- | --- |
| **COVID1A.** How serious of a problem do you think the coronavirus outbreak is for Haiti? **[Read alternatives]**  (1) Very serious (2) Somewhat serious (3) Not so serious (4) Not serious at all  (5) Have not thought much about this  (888888) **[DON’T READ]** Don’t know (988888) **[DON’T READ]** No answer  (999999) **[DON’T READ]** Inapplicable |  |

| **QUESTIONNAIRE A** | | | | | | | |
| --- | --- | --- | --- | --- | --- | --- | --- |
|  | **Very worried** | **Somewhat worried** | **A little worried** | **Not at all worried** | **[DON’T READ]**  **Don´t know** | **[DON’T READ]**  **No answer** | **[DON’T READ]**  **Inapplicable** |
| **COVID2AA**. How worried are you about the possibility that you or someone in your household will become sick with the coronavirus? **[Read alternatives]** | 1 | 2 | 3 | 4 | 888888 | 988888 | 999999 |
| **COVID2BA**. How worried are you about the possibility that your **household** economic situation will be negatively affected by the coronavirus? **[Read alternatives]** | 1 | 2 | 3 | 4 | 888888 | 988888 | 999999 |
| **COVID2CA**. How worried are you about the possibility that your **household** will have difficulty accessing basic goods, such as food or medicine, due to the coronavirus? **[Read alternatives]** | 1 | 2 | 3 | 4 | 888888 | 988888 | 999999 |

| **QUESTIONNAIRE A** | |
| --- | --- |
| **COVID3A.** How would you rate the performance of the national government in handling the coronavirus outbreak?  (1) Very good (2) Good (3) Neither good nor bad (fair) (4) Bad (5) Very bad (888888) **[DON’T READ]** Don´t know (988888) **[DON’T READ]** No answer  (999999) **[DON’T READ]** Inapplicable |  |
| **COVID4A.** Coronavirus is spreading in Haiti. Who is **the most responsible** for the increased number of infections? **[DO NOT READ THE LIST, MARK ONLY ONE RESPONSE]**  (1) The current administration  (2) Ourselves, the Haitian people  (3) The rich people of our country  (4) The United States  (5) China  (6) No one is to blame  (7) God/God’s will  (77) Other  (888888) **[DON’T READ]** Don´t know (988888) **[DON’T READ]** No answer  (999999) **[DON’T READ]** Inapplicable |  |

| **QUESTIONNAIRE A** | |
| --- | --- |
| Please tell me if you have taken any of the following actions because of the recent coronavirus outbreak. **[Randomize items]** | |
| **COVID6A.** Have you canceled any plans to attend large gatherings such as social events or church?  (1) Yes (2) No  (888888) **[DON’T READ]** Don’t know (988888) **[DON’T READ]** No answer  (999999) **[DON’T READ]** Inapplicable |  |
| **COVID7A.** Have you kept a greater distance between you and others when out in public?  (1) Yes (2) No  (888888) **[DON’T READ]** Don’t know (988888) **[DON’T READ]** No answer  (999999) **[DON’T READ]** Inapplicable |  |
| **COVID8A.** Have you washed your hands more often with water and soap or sanitizer?  (1) Yes (2) No  (888888) **[DON’T READ]** Don’t know (988888) **[DON’T READ]** No answer  (999999) **[DON’T READ]** Inapplicable |  |

| **QUESTIONNAIRE A** |  |
| --- | --- |
| **COVID14A.** Considering those who get the coronavirus, would you say that they deserve to be sick because of the way they live, or that they could not help becoming sick?  (1) Yes, they deserve to be sick (2) No, they could not help becoming sick  (888888) **[DON’T READ]** Don’t know (988888) **[DON’T READ]** No answer  (999999) **[DON’T READ]** Inapplicable |  |

Let´s talk now about the economy.

| **SOCT2.** Do you think that **the country’s** current economic situation is better than, the same as or worse than it was **12 months ago**?  (1) Better (2) Same (3) Worse  (888888) Don’t know **[DON’T READ]** (988888) No answer **[DON’T READ]** |  |
| --- | --- |
| **IDIO2.** Do you think that **your** economic situation is better than, the same as, or worse than it was **12 months** ago?  (1) Better (2) Same (3) Worse  (888888) Don’t know **[DON’T READ]** (988888) No answer **[DON’T READ]** |  |

| Now let’s talk about some services in Haiti… | | | | | | | |
| --- | --- | --- | --- | --- | --- | --- | --- |
| In general, how would you rate the quality of each of the following services in Haiti? Very good, good, neither good nor bad, bad or very bad? | **Very good** | **Good** | **Neither good nor bad** | **Bad** | **Very bad** | Don’t know **[DON’T READ]** | No answe**r [DON’T READ]** |
| **HAIACS5.** Drinkable water. Would you say that the service is... **[Read alternatives]** | 1 | 2 | 3 | 4 | 5 | 888888 | 988888 |
| **HAIACS6.** Trash disposal. Would you say that the service is... **[Read alternatives]** | 1 | 2 | 3 | 4 | 5 | 888888 | 988888 |

| **QUESTIONNAIRE A** |  |
| --- | --- |
| **ANESGI1.** Do you think what happens generally to people in your neighborhood will have something to do with what happens in your life?  (1) Yes (2) No  (888888) Don’t know **[DON’T READ]** (988888) No answer **[DON’T READ]**  (999999) **[DON’T READ]** Inapplicable |  |
| **IT1N1**. And speaking of the people from your neighborhood, would you say that people in your community are very trustworthy, somewhat trustworthy, not very trustworthy or untrustworthy...?  (1) Very trustworthy (2) Somewhat trustworthy  (3) Not very trustworthy (4) Untrustworthy  (888888) Don’t know **[DON’T READ]** (988888) No answer **[DON’T READ]**  (999999) **[DON’T READ]** Inapplicable |  |

| **QUESTIONNAIRE B** |  |
| --- | --- |
| **ANESGI2.** Do you think what happens generally to Haitians in this country will have something to do with what happens in your life?  (1) Yes (2) No  (888888) Don’t know **[DON’T READ]** (988888) No answer **[DON’T READ]**  (999999) **[DON’T READ]** Inapplicable |  |
| **IT1N2**. And speaking of Haitians, would you say that people in this country are very trustworthy, somewhat trustworthy, not very trustworthy or untrustworthy...?  (1) Very trustworthy (2) Somewhat trustworthy  (3) Not very trustworthy (4) Untrustworthy  (888888) Don’t know **[DON’T READ]** (988888) No answer **[DON’T READ]**  (999999) **[DON’T READ]** Inapplicable |  |

| **QUESTIONNAIRE A**  Now, changing the subject. Some people say that under some circumstances it would be justified for the Police Nationale d’Haïti to take power by a coup d’état (military coup). In your opinion would a coup by the police be justified…**[Read alternatives]**: | | | | | |
| --- | --- | --- | --- | --- | --- |
| **JC10**. When there is a lot of crime. | (1) A take-over by the police of the state would be justified | (2) A take-over by the police of the state would not be justified | Don’t know  **[DON’T READ]**  (888888) | No answer **[DON’T READ]**  (988888) | Inapplicable  **[DON’T READ]**  (999999) |

| **QUESTIONNAIRE B**  Now, changing the subject. Some people say that under some circumstances it would be justified for the Police Nationale d’Haïti to take power by a coup d’état (military coup). In your opinion would a coup by the police be justified…**[Read alternatives]**: | | | | | |
| --- | --- | --- | --- | --- | --- |
| **JC13**. When there is a lot of corruption. | (1) A take-over by the police of the state would be justified | (2) A take-over by the police of the state would not be justified | Don’t know  **[DON’T READ]**  (888888) | No answer **[DON’T READ]**  (988888) | Inapplicable  **[DON’T READ]**  (999999) |

| **JC13COVID.** When there is a public health emergency like the coronavirus | (1) A take-over by the police of the state would be justified | (2) A take-over by the police of the state would not be justified | Don’t know  **[DON’T READ]**  (888888) | No answer **[DON’T READ]**  (988888) |
| --- | --- | --- | --- | --- |

| **QUESTIONNAIRE A** | | | | | |
| --- | --- | --- | --- | --- | --- |
| **JCCOV1.** Do you believe that when there is a public health emergency like the coronavirus it is justifiable for the president of the country to postpone elections? | (1) Yes, it is justified | (2) No, it is not justified | Don’t know  **[DON’T READ]**  (888888) | No answer  **[DON’T READ]**  (988888) | Inapplicable  **[DON’T READ]**  (999999) |

| **QUESTIONNAIRE B** | | | | | |
| --- | --- | --- | --- | --- | --- |
| **JCCOV2.** Do you believe that when there is a lot of violence it is justifiable for the president of the country to postpone elections? | (1) Yes, it is justified | (2) No, it is not justified | Don’t know  **[DON’T READ]**  (888888) | No answer  **[DON’T READ]**  (988888) | Inapplicable  **[DON’T READ]**  (999999) |

| **VIC1EXT**. Now, changing the subject, have you been a victim of any type of crime in the past 12 months? That is, have you been a victim of robbery, burglary, assault, fraud, blackmail, extortion, violent threats or **any other type** of crime in the past 12 months?  (1) Yes (2) No  (888888) Don’t know **[DON’T READ]**  (988888) No answer **[DON’T READ]** |  |
| --- | --- |

| **AOJ11**. Speaking of the neighborhood where you live and thinking of the possibility of being assaulted or robbed, do you feel very safe, somewhat safe, somewhat **un**safe or very **un**safe?  (1) Very safe  (2) Somewhat safe  (3) Somewhat unsafe  (4) Very unsafe  (888888) Don’t know **[DON’T READ]** (988888) No answer **[DON’T READ]** |  |
| --- | --- |

| **IVOL24.** Is there a criminal gang or gangs in your neighborhood?  (0) No **[Skip to B0]**  (1) Yes **[Continue]**  (888888) Don’t know **[DON’T READ]** **[Skip to B0]**  (988888) No answer **[DON’T READ] [Skip to B0]** |
| --- |

| **GANG10.** In some places in this country, gangs are involved in a diverse set of activities. Would you say that gangs are involved in providing security for people in your neighborhood?  (1) Yes (2) No  (888888) Don’t know **[DON’T READ]** (988888) No answer **[DON’T READ]**  (999999) Inapplicable **[DON’T READ]** |
| --- |
| **GANG11.** How about providing services, such as helping people access basic goods like food. Would you say that gangs provide such services to people in your neighborhood?  (1) Yes (2) No  (888888) Don’t know **[DON’T READ]** (988888) No answer **[DON’T READ]**  (999999) Inapplicable **[DON’T READ]** |
| **GANG12.** How about acting as community leaders. Would you say that gangs act as community leaders for people in your neighborhood?  (1) Yes (2) No  (888888) Don’t know **[DON’T READ]** (988888) No answer **[DON’T READ]**  (999999) Inapplicable **[DON’T READ]** |

| **B0T.** I will ask you to answer the forthcoming questions using a number from a scale that goes from 1 to 7, where 1 is the lowest value, means NOT AT ALL and 7 the highest and means A LOT. If your opinion is between not at all and a lot, you would choose an intermediate score. To begin, to what extent do you like watching television? Tell me the number. **[Make sure that the respondent understands correctly]**. | | | | | | | | | | |
| --- | --- | --- | --- | --- | --- | --- | --- | --- | --- | --- |
| 1 | 2 | 3 | 4 | | 5 | 6 | 7 | 888888 | 988888 | |
| **Not at all** | | | | **A lot** | | | | **Don’t know**  **[DON’T READ]** | **No Answer**  **[DON’T READ]** | |
| **Note down a number 1-7, or 888888 Don´t know and 988888 No answer** | | | | | | | | | | |
| I am going to ask you a series of questions. I am going to ask that you use the numbers provided in the ladder to answer. Remember, you can use any number.  **B1**. To what extent do you think the courts in Haiti guarantee a fair trial? *[****Read:*** *If you think the courts do not ensure justice at all, choose number 1; if you think the courts ensure justice a lot, choose number 7, or choose a point in between the two.]* | | | | | | | | | |  |
| **B2**. To what extent do you respect the political institutions of Haiti? | | | | | | | | | |  |
| **B3**. To what extent do you think that citizens’ basic rights are well protected by the political system of Haiti? | | | | | | | | | |  |
| **B4**. To what extent do you feel proud of living under the political system of Haiti? | | | | | | | | | |  |
| **B6**. To what extent do you think that one should support the political system of Haiti? | | | | | | | | | |  |
| **BESS1.** To what extent do you think most people would try to take advantage of you if they got the chance? | | | | | | | | | |  |

| **M1**. Speaking in general of the current administration, how would you rate the job performance of President Jovenel Moïse? **[Read alternatives]**  (1) Very good (2) Good (3) Neither good nor bad (fair) (4) Bad (5) Very bad (888888) Don´t know **[DON’T READ]** (988888) No answer **[DON’T READ]** |  |
| --- | --- |

| **HAIM6.** Would you say that the election process in Haiti is very good, good, neither good nor bad, bad, or very bad?  (1) Very good (2) Good (3) Neither good nor bad (fair) (4) Bad (5) Very bad  (888888) **[DON’T READ]** Don’t know  (988888) **[DON’T READ]** No answer |  |
| --- | --- |

| For the next question I will ask you again to give me a response with a number. We will now use a scale that goes from 1, which means “strongly disagree” to 7 which means “strongly agree.” A number in between 1 and 7 represents an intermediate score. | | | | | | | | |
| --- | --- | --- | --- | --- | --- | --- | --- | --- |
| 1 | 2 | 3 | 4 | 5 | 6 | 7 | 888888 | 988888 |
| **Strongly disagree Strongly agree** | | | | | | | **Don’t know**  **[DON’T READ]** | **No answer**  **[DON’T READ]** |

**[Note down 1-7, 888888 = Don´t know, 988888=No answer]**

| **ING4.** Democracy may have problems, but it is better than any other form of government. To what extent do you agree or disagree with this statement? |  |
| --- | --- |

| **QUESTIONNAIRE A** | |
| --- | --- |
| **EXC7.** Taking into account your own experience or what you have heard, corruption among **public officials** is: **[Read alternatives]**  (1) Very common (2) Common (3) Uncommon  or (4) Very uncommon?  (888888) **[DON’T READ]** Don’t know  (988888) **[DON’T READ]** No answer  (999999) **[DON’T READ]** Inapplicable |  |

| **QUESTIONNAIRE B** | |
| --- | --- |
| **EXC7NEW.** Thinking of the politicians of Haiti… how many of them do you believe are involved in corruption? **[Read alternatives]**  (1) None  (2) Less than half of them  (3) Half of them  (4) More than half of them  (5) All  (888888) **[DON’T READ]** Don’t know  (988888) **[DON’T READ]** No answer  (999999) **[DON’T READ]** Inapplicable |  |

| **ESSH1.** How is your health in general? Would you say it is ... **[Read alternatives]**  (1) Very good (2) Good (3) Neither good nor bad (fair) (4) Bad (5) Very bad (888888) Don´t know **[DON’T READ]** (988888) No answer **[DON’T READ]** |  |
| --- | --- |

| **HC3.** Over the last 12 months that is from April 2019/May 2019 until today have you or any household member needed medical attention?  (1) Yes **[Continue]** (2) No **[Skip to SD6NEW2H]**  (888888) **[DON’T READ]** Don’t know **[Skip to SD6NEW2H]**  (988888) **[DON’T READ]** No answer **[Skip to SD6NEW2H]** |  |
| --- | --- |
| **HC4.** Referring to the last time you or any household member needed medical attention, who did you consult? **[Continue]**   1. General practitioner **[Continue]** 2. Specialist (doctor) **[Continue]** 3. Nurse **[Continue]** 4. Pharmacist **[Continue]** 5. Traditional healer (Doctor Fey) **[Continue]** 6. Houngan / mambo **[Continue]** 7. Relative/ Neighbor **[Continue]** 8. Other **[Continue]** 9. No consultation **[Skip to SD6NEW2H]**   (888888) **[DON’T READ]** Don’t know **[Skip to SD6NEW2H]**  (988888) **[DON’T READ]** No answer **[Skip to SD6NEW2H]**  (999999) **[DON’T READ]** Inapplicable |  |

| **HC5.** Where did you receive medical service? **[DO NOT read alternatives]**  (1) Private hospital **[Continue]**  (2) Public hospital **[Continue]**  (3) Community clinic (dispensaire) **[Continue]**  (4) Public health center **[Continue]**  (5) Private clinic **[Continue]**  (6) NGO clinic **[Continue]**  (7) The office of the person who performed the consultation **[Skip to SD6NEW2H]**  (8) Pharmacy **[Skip to SD6NEW2H]**  (9) At home **[Skip to SD6NEW2H]**  (10) Other **[Skip to SD6NEW2H]**  (888888) **[DON’T READ]** Don’t know  (988888) **[DON’T READ]** No answer  (999999) **[DON’T READ]** Inapplicable |  |
| --- | --- |
| **HC7.** How satisfied were you with the service at the health care facilities?  (1) Very satisfied (2) Somewhat satisfied  (3) Somewhat dissatisfied (4) Very dissatisfied  (888888) **[DON’T READ]** Don’t know  (988888) **[DON’T READ]** No answer  (999999) **[DON’T READ]** Inapplicable |  |

| **SD6NEW2H.** And thinking of the quality of public medical and health services in general? Are you…**[Read alternatives]**  (1) Very satisfied (2) Satisfied  (3) Dissatisfied (4) Very dissatisfied  (888888) Don´t know **[DON’T READ]** (988888) No answer **[DON’T READ]** (999999) Inapplicable (Does not use) **[DON’T READ]** |  |
| --- | --- |

| **QUESTIONNAIRE A** |  |
| --- | --- |
| **POL1A.** How much interest do you have in politics: a lot, some, little or none?  (1) A lot (2) Some (3) Little (4) None  (888888) Don’t know **[DON’T READ]** (988888) No answer **[DON’T READ]**  (999999) **[DON’T READ]** Inapplicable |  |
| **VB20A.** If the next presidential elections were being held this week, what would you do? **[Read alternatives]**  (1) Wouldn’t vote  (2) Would vote for the current (incumbent) candidate or party  (3) Would vote for a candidate or party different from the current administration  (4) Would go to vote but would leave the ballot blank or would purposely cancel my vote  (888888) Don’t know **[DON’T READ]** (988888) No answer **[DON’T READ]**  (999999) **[DON’T READ]** Inapplicable |  |

| **QUESTIONNAIRE B** |  |
| --- | --- |
| **VB20B.** If the next presidential elections were being held this week, what would you do? **[Read alternatives]**  (1) Wouldn’t vote  (2) Would vote for the current (incumbent) candidate or party  (3) Would vote for a candidate or party different from the current administration  (4) Would go to vote but would leave the ballot blank or would purposely cancel my vote  (888888) Don’t know **[DON’T READ]** (988888) No answer **[DON’T READ]**  (999999) **[DON’T READ]** Inapplicable |  |
| **POL1B.** How much interest do you have in politics: a lot, some, little or none?  (1) A lot (2) Some (3) Little (4) None  (888888) Don’t know **[DON’T READ]** (988888) No answer **[DON’T READ]**  (999999) **[DON’T READ]** Inapplicable |  |

| **QUESTIONNAIRE A** | | | | | | |
| --- | --- | --- | --- | --- | --- | --- |
| Changing the subject… | **More** | **Same** | **Less** | Don’t know **[DON’T READ]** | No answe**r [DON’T READ]** | Inapplicable  **[DON’T READ]** |
| **HAIRIG1.** Do you think that now you know more, about the same, or less about your rights than 12 months ago? | 1 | 2 | 3 | 888888 | 988888 | 999999 |

| **QUESTIONNAIRE B** | | | | | | |
| --- | --- | --- | --- | --- | --- | --- |
| Changing the subject… | **More** | **Same** | **Less** | Don’t know **[DON’T READ]** | No answer **[DON’T READ]** | Inapplicable  **[DON’T READ]** |
| **HAIRIG5.** Do you feel you know more, about the same, or less about your rights to access to justice than 12 months ago? | 1 | 2 | 3 | 888888 | 988888 | 999999 |

| **WF1.** Do you or someone in your household receive regular assistance in the form of money, food, or products from the government, not including pensions/social security?  (1) Yes (2) No  (888888) Don´t know **[DON’T READ]** (988888) No answer **[DON’T READ]** |  |
| --- | --- |

| **QUESTIONNAIRE B**  Now, we would like to ask you some questions about the coronavirus situation. | |
| --- | --- |
| **COVID1B.** How serious of a problem do you think the coronavirus outbreak is for Haiti? **[Read alternatives]**  (1) Very serious (2) Somewhat serious (3) Not so serious (4) Not serious at all  (5) Haven’t thought much about it  (888888) **[DON’T READ]** Don’t know (988888) **[DON’T READ]** No answer  (999999) **[DON’T READ]** Inapplicable |  |

| **QUESTIONNAIRE B** | | | | | | | |
| --- | --- | --- | --- | --- | --- | --- | --- |
|  | **Very worried** | **Somewhat worried** | **A little worried** | **Not at all worried** | **[DON’T READ]**  **Don´t know** | **[DON’T READ]**  **No answer** | **[DON’T READ]**  **Inapplicable** |
| **COVID2AB**. How worried are you about the possibility that you or someone in your household will become sick with the coronavirus? **[Read alternatives]** | 1 | 2 | 3 | 4 | 888888 | 988888 | 999999 |
| **COVID2BB**. How worried are you about the possibility that your **household** economic situation will be negatively affected by the coronavirus? **[Read alternatives]** | 1 | 2 | 3 | 4 | 888888 | 988888 | 999999 |
| **COVID2CB**. How worried are you about the possibility that your **household** will have difficulty accessing basic goods, such as food or medicine, due to the coronavirus? **[Read alternatives]** | 1 | 2 | 3 | 4 | 888888 | 988888 | 999999 |

| **QUESTIONNAIRE B** | |
| --- | --- |
| **COVID3B.** How would you rate the performance of the national government in handling the coronavirus outbreak?  (1) Very good (2) Good (3) Neither good nor bad (fair) (4) Bad (5) Very bad (888888) **[DON’T READ]** Don´t know (988888) **[DON’T READ]** No answer  (999999) **[DON’T READ]** Inapplicable |  |
| **COVID4B.** Coronavirus is spreading in Haiti. Who is the **most responsible** for the increased number of infections? **[DO NOT READ THE LIST, MARK ONLY ONE RESPONSE]**  (1) The current administration  (2) Ourselves, the Haitian people  (3) The rich people of our country  (4) The United States  (5) China  (6) No one is to blame  (7) God/God’s will  (77) Other  (888888) **[DON’T READ]** Don´t know (988888) **[DON’T READ]** No answer  (999999) **[DON’T READ]** Inapplicable |  |

| **QUESTIONNAIRE B**  Please tell me if you have taken any of the following actions because of the recent coronavirus outbreak. **[Randomize items]** | |
| --- | --- |
| **COVID6B.** Have you canceled any plans to attend large gatherings such as social events or church?  (1) Yes (2) No  (888888) **[DON’T READ]** Don’t know (988888) **[DON’T READ]** No answer  (999999) **[DON’T READ]** Inapplicable |  |
| **COVID7B.** Have you kept a greater distance between you and others when out in public?  (1) Yes (2) No  (888888) **[DON’T READ]** Don’t know (988888) **[DON’T READ]** No answer  (999999) **[DON’T READ]** Inapplicable |  |
| **COVID8B.** Have you washed your hands more often with water and soap or sanitizer?  (1) Yes (2) No  (888888) **[DON’T READ]** Don’t know (988888) **[DON’T READ]** No answer  (999999) **[DON’T READ]** Inapplicable |  |

| **QUESTIONNAIRE B** |  |
| --- | --- |
| **COVID14B.** Considering those who get the coronavirus, would you say that they deserve to be sick because of the way they live, or that they could not help becoming sick?  (1) Yes, they deserve to be sick (2) No, they could not help becoming sick  (888888) **[DON’T READ]** Don’t know (988888) **[DON’T READ]** No answer  (999999) **[DON’T READ]** Inapplicable |  |

| **ED**. How many years of schooling have you completed?  _____ Year ___________________ (primary, secondary, university) = ________ total number of years **[Use the table below for the code]** | |
| --- | --- |
| (0) 0 years | None |
| (1) 1 year | Pre-School |
| (2) 2 years | Preparatory1 / 1 A.F. |
| (3) 3 years | Preparatory 2 / 2 A.F. |
| (4) 4 years | Elementary 1 / 3 A.F. |
| (5) 5 years | Elementary 2 / 4 A.F. |
| (6) 6 years | Intermediate 1 / 5 A.F. |
| (7) 7 years | Intermediate 2 / 6 A.F. |
| (8) 8 years | Sixième / 7 A.F. |
| (9) 9 years | Cinquième / 8 A.F |
| (10) 10 years | Quatrième / 9 A.F. |
| (11) 11 years | Troisième |
| (12) 12 years | Seconde |
| (13) 13 years | Rhéto |
| (14) 14 years | Philo |
| (15) 15 years | University 1 |
| (16) 16 years | University 2 |
| (17) 17 years | University 3 |
| (18) 18 years | University 4 or more |
| (888888) | **[DON’T Read]** Don’t know |
| (988888) | **[DON’T Read]** No answer |

| **Q5B**. Could you please tell me: how important is religion in your life? **[Read alternatives]**  (1) Very important (2) Somewhat important (3) Not very important  (4) Not at all important (888888) Don’t know **[DON’T READ]**  (988888) Don’t answer **[DON’T READ]** |  |
| --- | --- |
| **OCUP4A.** How do you mainly spend your time? Are you currently **[Read alternatives]**  (1) Working?  (2) Not working, but have a job?  (3) Actively looking for a job?  (4) A student?  (5) Taking care of the home?  (6) Retired, a pensioner or permanently disabled to work  (7) Not working and not looking for a job?  (888888) Don´t know **[DON’T READ]**  (988888) No answer **[DON’T READ]** |  |
| **Q10A.** Do you or someone else living in your household receive remittances (financial support), that is, economic assistance from abroad?  (1) Yes (2) No  (888888) Don’t know **[DON’T READ]** (988888) No answer **[DON’T READ]** |  |
| **Q14.** Do you have any intention of going to live or work in another country in the next three years? (1) Yes (2) No  (888888) Don’t know **[DON’T READ]**  (988888) No answer **[DON’T READ]** |  |
| **Q10E.** Over the past two years, has the income of your household: **[Read alternatives]**  (1) Increased?  (2) Remained the same?  (3) Decreased?  (888888) Don’t know **[DON’T READ]**  (988888) No answer **[DON’T READ]** |  |

| Now I am going to read you some questions about food. | | | | | |
| --- | --- | --- | --- | --- | --- |
|  | **No** | **Yes** | Don’t know  **[DON’T READ]** | No answer  **[DON’T READ]** |  |
| **FS2.** In the past three months, because of a lack of money or other resources, did your household ever run out of food? | 0  **[Skip to GI0N]** | 1  **[Continue]** | 888888  **[Skip to GI0N]** | 988888 **[Skip to GI0N]** |  |
| **FS2COVID.** And did this happen because of the coronavirus or for another reason?  (1) Coronavirus  (2) Another reason  (888888) Don’t know **[DON’T READ]**  (988888) No answer **[DON’T READ]**  (999999) **[DON’T Read]** Inapplicable | | | | | |

| **GI0N.** About how often do you pay attention to the news, whether on TV, the radio, newspapers or the internet? **[Read alternatives]:**  (1) Daily (2) A few times a week (3) A few times a month  (4) A few times a year (5) Never  (888888) Don’t know **[DON’T READ]** (988888) No answer **[DON’T READ]** |  |
| --- | --- |

Could you tell me if you have the following in your house: **[Read out all items]**

| **R3**. Refrigerator | (0) No | | | (1) Yes | Don’t know  **[DON’T READ]**  (888888) | No answer  **[DON’T READ]**  (988888) | |
| --- | --- | --- | --- | --- | --- | --- | --- |
| **R4.** Landline/residential telephone (not cellular) | (0) No | | | (1) Yes | Don’t know  **[DON’T READ]**  (888888) | No answer  **[DON’T READ]**  (988888) | |
| **R4A**. Cellular telephone/mobile. (Accept smartphone) | (0) No | | | (1) Yes | Don’t know  **[DON’T READ]**  (888888) | No answer  **[DON’T READ]**  (988888) | |
| **R5**. Vehicle/car. How many? **[If the interviewee does not say how many, mark “one.”]** | (0) No | (1) One | (2) Two | (3) Three or more | Don’t know  **[DON’T READ]**  (888888) | No answer  **[DON’T READ]**  (988888) | |
| **R6**. Washing machine | (0) No | | | (1) Yes | Don’t know  **[DON’T READ]**  (888888) | No answer  **[DON’T READ]**  (988888) | |
| **R7**. Microwave oven | (0) No | | | (1) Yes | Don’t know  **[DON’T READ]**  (888888) | No answer  **[DON’T READ]**  (988888) | |
| **R8**. Motorcycle | (0) No | | | (1) Yes | Don’t know  **[DON’T READ]**  (888888) | No answer  **[DON’T READ]**  (988888) | |
| **R12**. Drinking water line/pipe to the house | (0) No | | | (1) Yes | Don’t know  **[DON’T READ]**  (888888) | No answer  **[DON’T READ]**  (988888) | |
| **R14**. Indoor bathroom/toilet/WC | (0) No | | | (1) Yes | Don’t know  **[DON’T READ]**  (888888) | No answer  **[DON’T READ]**  (988888) | |
| **R15.** Computer (Accept tablet, iPad) | (0) No | | | (1) Yes | Don’t know  **[DON’T READ]**  (888888) | No answer  **[DON’T READ]**  (988888) | |
| **R18.** Internet from your home (included phone or tablet) | (0) No | | | (1) Yes | Don’t know  **[DON’T READ]**  (888888) | No answer  **[DON’T READ]**  (988888) | |
| **R1**. Television | (0) No **[Skip to PSC1]** | | | (1) Yes **[Continue]** | Don’t know  **[DON’T READ]**  (888888) | No answer  **[DON’T READ]**  (988888) | |
| **R16.** Flat panel TV | (0) No | | | (1) Yes | Don’t know  **[DON’T READ]**  (888888) | No answer  **[DON’T READ]**  (988888) | **Inapplicable**  **[DON’T READ]**  (999999) |

| **PSC1.** What is the **main** source of **drinking-water** for members of your household? **[DO NOT read alternatives. Mark only one answer] [If respondent mentions more than one source, ask for the one most used]**  **[If respondent only says piped/tap water or public water probe or check if the connection goes (1) inside the household or (2) outside the household]**  (01) Piped water/public water pipe/tap water into dwelling/house  (02) Piped water to yard/plot  (03) Irregular connection (stealing) to public water pipe  (04) Public tap/ standpipe /tank  (05) Tubewell/borehole (with pump)  (06) Protected dug well (without pump)  (07) Unprotected dug well (without pump)  (08) Protected spring  (09) Unprotected spring  (10) Rainwater collection  (11) Bottled water (water bags)  (12) Cart with small tank/drum  (13) Tanker-truck  (14) River, creek, stream, canal, irrigation channels  (77) Other  (888888) **[DON’T READ]** Don’t know  (988888) **[DON’T READ]** No answer |  |
| --- | --- |
| **PSC2.** What is the main source of water used by your household for other purposes, such as **cooking and hand washing**? **[DO NOT read alternatives. Mark only one answer.] [If respondent mentions more than one source, ask for the one most used]**  **[If respondent only says piped/tap water or public water probe or check if the connection goes (1) inside the household or (2) outside the household]**  (01) Piped water/public water pipe/tap water into dwelling/home **[Continue]**  (02) Piped water to yard/plot **[Continue]**  (03) Irregular connection (stealing) to public water pipe **[Continue]**  (04) Public tap/standpipe/tank **[Skip to PSC11]**  (05) Tubewell/borehole (with pump) **[Skip to PSC11]**  (06) Protected dug well (without pump) **[Skip to PSC11]**  (07) Unprotected dug well (without pump) **[Skip to PSC11]**  (08) Protected spring **[Skip to PSC11]**  (09) Unprotected spring **[Skip to PSC11]**  (10) Rainwater collection **[Skip to PSC11]**  (11) Bottled water (water bags) **[Skip to PSC11]**  (12) Cart with small tank/drum **[Skip to PSC11]**  (13) Tanker-truck **[Skip to PSC11]**  (14) River, creek, stream, canal, irrigation channels **[Skip to PSC11]**  (77) Other **[Skip to PSC11]**  (888888) **[DON’T READ]** Don’t know **[Skip to PSC11]**  (988888) **[DON’T READ]** No answer **[Skip to PSC11]** |  |
| **[Ask only if there is water piping– If answer to PSC2 was (01) (02) or (03)]**  **PSC7.** How many days a week do you receive water from the water piping/public water line?  (0) Less than once a week  (1) One day a week  (2) Two days per week  (3) Three days per week  (4) Four days per week  (5) Five days per week  (6) Six days per week  (7) Seven days per week  (888888) **[DON’T READ]** Don’t know  (988888) **[DON’T READ]** No answer  (999999) **[DON’T READ]** Inapplicable |  |
| **[Ask only if there is water piping– If answer to PSC2 was (01) (02) or (03)]**  **PSC8.** How many hours of water per day do you get during the days you have service?  Write down amount of hours____________________________ **[Attention, if time reported in minutes or fractions of hours, round up to the hour]**  **[It refers to supply through the pipeline, with independence from the home storage system they might have]**  **[Maximum value accepted: 24]**  (888888) **[DON’T READ]** Don’t know  (988888) **[DON’T READ]** No answer  (999999) **[DON’T READ]** Inapplicable |  |
| **[Ask only if there is water piping– If answer to PSC2 was (01) (02) or (03)]**  **PSC9.** During the past four weeks, how many times has the regular water service been interrupted? **[Write down how many times]** __________________  **[Maximum value accepted: 50]**  (888888) **[DON’T READ]** Don’t know  (988888) **[DON’T READ]** No answer  (999999) **[DON’T READ]** Inapplicable |  |
| **PSC11.** The bathroom or toilet facility/sanitary in this household is connected to… **[Read alternatives]** **[Probe if necessary. Attention: Options (1) to (5) and (7) imply connection to a system or discharge outside the household]**  (1) Piped sewer system **[Skip to PSC12]**  (7) Connected to treatment plant/system **[Skip to PSC12]**  (2) Septic tank/hole **outside** the house **[Skip to PSC12]**  (3) Tubing flowing to creek/waterway **[Skip to PSC12]**  (4) Other [flows/discharges somewhere else] **[Skip to PSC12]**  (5) Flows/discharges to unknown place/not sure/does not know where **[Skip to PSC12]**  (6) Cesspit not connected to any system **[Continue]**  (888888) **[DON’T READ]** Don’t know **[Skip to PSC12]**  (988888) **[DON’T READ]** No answer **[Skip to PSC12]**  **[NOTE: Option (7) was not included in Colombia, Costa Rica and Honduras]** |  |
| **[Ask only if not connected to any system, response (6) in PSC11]**  **PSC11A.** What do you use in your household as a bathroom? **[Read alternatives]**  (1) Ventilated pit latrine  (2) Pit latrine with slab/toilet  (3) Pit latrine without slab/toilet/open pit  (4) Composting toilet/ dry toilet/ eco toilet  (5) Bucket  (6) Hanging toilet/hanging latrine  (7) No facilities or uses bush or field  (77) Other  (888888) **[DON’T READ]** Don’t know  (988888) **[DON’T READ]** No answer  (999999) **[DON’T READ]** Inapplicable |  |
| **PSC12.** Do you share this facility with other households?  (1) Yes (2) No  (888888) **[DON’T READ]** Don’t know  (988888) **[DON’T READ]** No answer |  |
| **PSC13.** Please, can you tell me how do you dispose of the garbage in this household?  **[DO NOT read alternatives. Mark all that apply] [Probe to find out if household waste collection is formal (01) or informal (02), and for recycling if formal (12) or informal (13)]**  (01) Household waste collection (municipal/formal service)  (02) Household waste collection (informal service/garbage pickers)  (03) Disposal at neighborhood/community waste containers  (04) Take it to municipal landfill  (05) Bury it  (06) Make fertilizer/compost  (07) Burn it  (08) Disposal in vacant lot/waste land or in waterway  (09) Disposal in other/any place  (10) Recycle at home (not compost)  (11) Take it to recycling center  (12) Household recycling collection (formal/municipal)  (13) Household recycling collection (informal/garbage pickers)  (888888) **[DON’T READ]** Don’t know  (988888) **[DON’T READ]** No answer |  |
| **PSC3.** I am going to mention some problems many of us Haitians have faced in the last years. Which ones of these have you personally, or someone from your household, experienced in the last three years? **[Read alternatives. Mark all that apply]**  (0) **[DON’T READ]** None  (1) Droughts that have as a consequence water outages or lack of water  (2) Electricity outages  (3) Floods  (888888) **[DON’T READ]** Don’t know  (988888) **[DON’T READ]** No answer |  |
| **PSC4.** Who do you think is primarily responsible for droughts that have as a consequence water outages or lack of water? **[Read alternatives]**  (1) National/central government  (3) Local/ municipal government  (4) Water service company/DINEPA  (5) Climate change or extreme weather conditions  (6) People/ourselves  (77) Other  (888888) **[DON’T READ]** Don’t know  (988888) **[DON’T READ]** No answer |  |
| **PSC5.** Who do you think is primarily responsible for the electricity outages? **[Read alternatives]**  (1) National/central government  (3) Local/municipal government  (4) Electricity company/[company name]  (5) Climate change or extreme weather conditions  (6) People/ourselves  (77) Other  (888888) **[DON’T READ]** Don’t know  (988888) **[DON’T READ]** No answer |  |
| **PSC6.** Who do you think is primarily responsible for the floods? **[Read alternatives]**  (1) National/central government  (3) Local/municipal government  (5) Climate change or extreme weather conditions  (6) People/ourselves  (77) Other  (888888) **[DON’T READ]** Don’t know  (988888) **[DON’T READ]** No answer |  |

| **LINEA1.** Are you the owner of this telephone line?  (1) Yes (2) No  (888888) **[DON’T READ]** Don’t know  (988888) **[DON’T READ]** No answer |  |
| --- | --- |
| **LINEA2.** Is this a single-user telephone line or is it shared among various users?  (1) Single user telephone line  (2) Share among various users  (888888) **[DON’T READ]** Don’t know  (988888) **[DON’T READ]** No answer |  |
| **LINEA3.** How many cellphone lines do you possess? Tell me just the number of cellphone lines under your name _____________  (888888) **[DON’T READ]** Don’t know  (988888) **[DON’T READ]** No answer |  |

***These are all the questions I have. Thank you very much for your cooperation.***

| **[When the interview is complete, WITHOUT asking, please complete the following questions]** |
| --- |
| **NOISE1T.** Was there anyone else present during the interview besides the interviewee?  (0) No  (1) Yes, but at a distance. Does not interfere with the call  (3) Yes, interfering/participating sporadically  (4) Yes, interfering a great deal/jeopardizing the interview |
| **CONOCIM.** Using the scale shown below, please rate your perception about the level of political knowledge of the interviewee.  (1) Very high (2) High (3) Neither high or low (4) Low (5) Very low |

| **TI**. Duration of interview ***[minutes, see page # 1]*** _____________ |  |
| --- | --- |
| **INTID.** Interviewer ID number: ____________ |  |
| **SEXI. Note interviewer’s sex:** (1) Male (2) Female |  |

| *I swear that this interview was carried out with the person indicated above.* |
| --- |
| *Interviewer’s signature__________________ Date ____ /_____ /_____*  *Field supervisor’s signature _______________________________________* |
| *Comments: ________________________________________________________________________ __________________________________________________________________________________* |
| *[Not for PDA/Android use] Signature of the person who entered the data _______________________* |
| *[Not for PDA/Android use] Signature of the person who verified the data _______________________* |

**6. Complete survey questionnaire (Haitian Creole)**

*AmericasBarometer 2018/19 Haiti Questionnaire Version # 12.0.10.0.* ***IRB Approval #:*** *170509*

|  | | |
| --- | --- | --- |
|  |  |  |

**LAPOP: Ayiti, 2020**

© Vanderbilt University 2020. All rights reserved.

| **PAIS. Country**:   \| 01. Meksik \| 02. Gwatemala \| 03. Salvadò \| 04. Ondiras \| 05. Nikaragwa \| \| --- \| --- \| --- \| --- \| --- \| \| 06. Kosta Rika \| 07. Panama \| 08. Kolonbi \| 09. Ekwatè \| 10. Bolivi \| \| 11. Pewou \| 12. Paragwey \| 13. Chili \| 14. Irigwey \| 15. Brezil \| \| 16. Venezyela \| 17. Ajantin \| 21. Dominikani \| **22. Ayiti** \| 23. Jamayik \| \| 24. Giyànn \| 25. Trinidad \| 26. Beliz \| 40. Letazini \| 41. Kanada \| \| 27. Sirinam \| 28. Baamas \| 29. Babad \|  \|  \| |  |
| --- | --- | --- | --- | --- | --- | --- | --- | --- | --- | --- | --- | --- | --- | --- | --- | --- | --- | --- | --- | --- | --- | --- | --- | --- | --- | --- | --- | --- | --- | --- | --- |
| **IDNUM.** Nimewo Kèksyonè a **[Pou ekri nan biwo a]** |  |
| **Q2.** Ki laj ou? **[Nòt: EKRI LAJ MOUN KI AP BA OU ANTREVI A]** |  |
| **CIUDADANO1T.** Pou Kòmanse, èske w se sitwayen ayisyen oswa ou reside tout tan an Ayiti?  (1) Wi (2) Non |  |
| **Q1T.** Èske ou ka dim ki sèks ou?  (1) Gason (2) Fanm (3) Lòt |  |
| **PROV1T.** Nan ki depatman wap viv? _______________ |  |
| **MUNICIPIO1T.** Nan ki komin nan depatman {non depatman} wap viv? ___________ |  |
| **UR1.** Èske w abite nan zòn iben (nan vil) nan komin lan, oswa nan zòn riral (an deyò) nan komin lan?  (1) Zòn iben  (2) Zòn riral  (888888) **[PA LI REPONS LA]** Pa konnen  (988888) **[PA LI REPONS LA]** Pa reponn |  |
| **IDIOMAQ. Lang ankèt la:** (14) Kreyòl |  |
| **Lè ak minit ankèt la kòmanse:** _____:_____ |  |
| **FÈCHA. Dat ankèt la Jou**: ____ **Mwa:_______**  Lane: 2020 |  |
| **REMAK: [PA LI] SE POU W TOUJOU LI FÉY ENFÒMASYON ETID LA POU MOUN YO. ANPLIS SE POU W TANN MOUN NAN REPONN OU SI LI DAKÒ OSINON SI LI PA DAKÒ FÉ ENTÉVYOU A AVAN OU KOMANSE.** | |

| **KESYONE** **A**  Pou komanse, nou ta renmen poze ou kèk kesyon sou sityasyon kowonaviris la. | |
| --- | --- |
| **COVID1A.** Nan ki nivo ou panse ke epidemi kowonaviris la se yon pwoblèm ki grav pou Ayiti? **[Li repons yo]**  (1) Li grav anpil (2) Ontijan grav (3) Li pa si grav (4) Li pa grav ditou  (5) M pa panse anpil ak sa  (888888) **[PA LI REPONS LA]** Pa konnen (988888) **[PA LI REPONS LA]** Pa reponn  (999999) **[PA LI REPONS LA]** Pa aplike |  |

| **KESYONE** **A** | | | | | | | |
| --- | --- | --- | --- | --- | --- | --- | --- |
|  | **Preokipe anpil** | **Ontijan preokipe** | **On ti kras preokipe** | **Pa preokipe ditou** | **[PA LI REPONS LA]**  **Pa konnen** | **[PA LI REPONS LA]**  **Pa reponn** | **[PA LI REPONS LA]**  **Pa aplike** |
| **COVID2AA**. Nan ki nivo ou preokipe ke ou menm oswa yon moun kap viv nan kay sa a ta ka vin malad ak kowonaviris? **[Li repons yo]** | 1 | 2 | 3 | 4 | 888888 | 988888 | 999999 |
| **COVID2BA**. Nan ki nivo ou preokipe ke sityasyon ekonomik moun kap viv nan kay sa a kapab afekte negativman pa kowonaviris la? **[Li repons yo]** | 1 | 2 | 3 | 4 | 888888 | 988888 | 999999 |
| **COVID2CA**. Nan ki nivo ou preokipe ke moun kap viv nan kay sa a kapab gen difikilte poul jwenn bagay bazik tankou manje, medikaman akoz kowonaviris la? **[Li repons yo]** | 1 | 2 | 3 | 4 | 888888 | 988888 | 999999 |

| **KESYONE A** | |
| --- | --- |
| **COVID3A.** Kouman ou ta evalye pèfòmans gouvènman santral la nan fason lap fè fas ak epidemi kowonaviris la?  (1) Li bon anpil (2) Li bon (3) Pa pi mal (4) Li mal (5) Li mal anpil  (888888) **[PA LI REPONS LA]** Pa konnen  (988888) **[PA LI REPONS LA]** Pa reponn  (999999) **[PA LI REPONS LA]** Pa aplike |  |
| **COVID4A.** Kowonaviris la ap layite kol an Ayiti, kiyès ou panse **ki gen** **plis responsabilite** pou kantite ka kap ogmante yo? **[PA LI LIST REPONS SA YO. EKRI SELMAN YON SEL REPONS]**  (1) Gouvènman sa a  (2) Nou menm, pèp ayisyen  (3) Moun ki gen lajan, boujwa peyi a  (4) Etazini  (5) La chinn  (6) Okenn moun pa gen tò nan sa  (7) Se bondye/se volontel  (77) Lòt repons  (888888) **[PA LI REPONS LA]** Pa konnen  (988888) **[PA LI REPONS LA]** Pa reponn  (999999) **[PA LI REPONS LA]** Pa aplike |  |

| **KESYONE A**  Èske ou ka dim si ou te pran youn nan desizyon sayo akoz de epidemi kowonaviris la? **[Randomize items]** | |
| --- | --- |
| **COVID6A.** Eske ou te anile plan pou patisipe nan reyinyon ki ta pral gen anpil moun tankou evènman sosyal, al legliz?  (1) Wi (2) Non  (888888) **[PA LI REPONS LA]** Pa konnen (988888) **[PA LI REPONS LA]** Pa reponn  (999999) **[PA LI REPONS LA]** Pa aplike |  |
| **COVID7A.** Èske w te kenbe pi gwo distans ant ou menm ak lòt moun lè w sòti nan lari?  (1) Wi (2) Non  (888888) **[PA LI REPONS LA]** Pa konnen (988888) **[PA LI REPONS LA]** Pa reponn  (999999) **[PA LI REPONS LA]** Pa aplike |  |
| **COVID8A.** Èske w lave men ou pi souvan avèk dlo, savon oswa dezenfektan?  (1) Wi (2) Non  (888888) **[PA LI REPONS LA]** Pa konnen (988888) **[PA LI REPONS LA]** Pa reponn  (999999) **[PA LI REPONS LA]** Pa aplike |  |

| **KESYONE A** |  |
| --- | --- |
| **COVID14A.** Lè ou konsidere moun ki gen kowonaviris la, èske ou ta di ke yo merite maladi a akoz de jan yo menen vi yo, oswa yo pa gen oken responsabilite nan sa?  (1) Wi yo merite li (2) Non yo pa gen okenn responsabilite nan maladi a  (888888) **[PA LI REPONS LA]** Pa konnen (988888) **[PA LI REPONS LA]** Pa reponn  (999999) **[PA LI REPONS LA]** Pa aplike |  |

Ann pale de ekonomi an.

| **SOCT2.** Eske w konsidere sitiyasyon ekonomik **peyi a** nan moman sa a vin: pi bon, menm jan oswa pi mal pa rapò a **12 mwa ki fèk sot pase yo**?  (1) Pi bon (2) Menn jan (3) Pi mal  (888888) Pa konnen **[PA LI REPONS LA]**  (988888) Pa reponn **[PA LI REPONS LA]** |  |
| --- | --- |
| **IDIO2.** Eske w konsidere **pwòp** sitiyasyon ekonomik pa w nan moman sa a pi bon, menm jan oswa pi mal pa rapò a 12 mwa ki fèk sot pase yo?  (1) Pi bon (2) Menn jan (3) Pi mal  (888888) Pa konnen **[PA LI REPONS LA]** (988888) Pa reponn **[PA LI REPONS LA]** |  |

| Kounya, ann pale de kèk sèvis an Ayiti | | | | | | | |
| --- | --- | --- | --- | --- | --- | --- | --- |
| Jeneralman, kijan w ta di sèvis sa yo ye nan peyi Dayiti? Ou ta di: yo pi bon, yo bon, yo pa pi mal, yo mal osinon yo pi mal? | **Li pi bon** | **Li bon** | **Li pa pi mal** | **Li**  **Mal** | **Li Pi mal** | Pa konnen **[PA LI REPONS LA]** | Pa reponn **[PA LI REPONS LA]** |
| **HAIACS5.** Dlo potab. Ou ta di sèvis la… **[Li repons yo]** | 1 | 2 | 3 | 4 | 5 | 888888 | 988888 |
| **HAIACS6.** Ranmase fatra. Ou ta di sèvis la… **[Li repons yo]** | 1 | 2 | 3 | 4 | 5 | 888888 | 988888 |

| **KESYONE A** |  |
| --- | --- |
| **ANESGI1.** Eske ou panse sa ki kon rive moun nan zòn kotew rete a, ka gen pou wè ak sa ki ka rivew nan la vi?  (1) Wi (2) Non  (888888) Pa konnen **[PA LI REPONS LA]** (988888) Pa reponn **[PA LI REPONS LA ]** (999999) Pa aplike **[PA LI REPONS LA]** |  |
| **IT1N1.** An palan de moun nan katye kote w rete, eske w ka di m si w genyen anpil konfyans, konfyans, pa anpil konfyans, osinon ankenn konfyans nan moun nan zòn nan…?  (1) Anpil konfyans (2) Konfyans  (3) Pa anpil konfyans (4) Ankenn konfyans  (888888) Pa konnen **[PA LI REPONS LA]** (988888) Pa reponn **[PA LI REPONS LA]** (999999) **[DON’T READ]** Inapplicable  (999999) Pa aplike **[PA LI REPONS LA]** |  |

| **KESYONE B** |  |
| --- | --- |
| **ANESGI2.** Eske ou panse sa ki kon rive ayisyen nan peyi a, ka gen pou wè ak sa ki ka rivew nan la vi?  (1) Wi (2) Non  (888888) Pa konnen **[PA LI REPONS]** (988888) Pa reponn **[PA LI REPONS LA ]**  (999999) Paaplike **[PA LI REPONS LA]** |  |
| **IT1N2.** Kounya, ann pale de Ayisyen, eske w ka di m si w genyen anpil konfyans, konfyans, pa anpil konfyans, osinon ankenn konfyans nan moun kap viv nan peyi a…?  (1) Anpil konfyans (2) Konfyans  (3) Pa anpil konfyans (4) Ankenn konfyans  (888888) Pa konnen **[PA LI REPONS LA]** (988888) Pa reponn **[PA LI REPONS LA]** (999999) **[DON’T READ]** Inapplicable  (999999) Pa aplike **[PA LI REPONS LA]** |  |

| **KESYONE A**  Konnye a, ann pale lòt bagay. Gen kèk moun ki di nan kèk sikonstans, ta gen rezon sifizan pou lapolis nasyonal pran pouvwa a pa yon koudeta. Daprè ou menm, èske ta gen rezon pou lapolis bay yon koudeta nan sikonstans sa yo? **[Li repons yo]**: | | | | | |
| --- | --- | --- | --- | --- | --- |
| **JC10**. Lè gen anpil krim. | (1) Li ta gen rezon sifizan pou lapolis pran pouvwa a | (2) Li pa ta gen rezon sifizan pou lapolis pran pouvwa a | Pa konnen  **[PA LI REPONS LA]** (888888) | Pa reponn  **[PA LI REPONS LA]**  (988888) | Pa aplike  **[PA LI REPONS LA]**  (999999) |

| **KESYONE B**  Konnye a, ann pale lòt bagay. Gen kèk moun ki di nan kèk sikonstans, ta gen rezon sifizan pou lapolis nasyonal pran pouvwa a pa yon koudeta. Daprè ou menm, èske ta gen rezon pou lapolis bay yon koudeta nan sikonstans sa yo? **[Li repons yo]**: | | | | | |
| --- | --- | --- | --- | --- | --- |
| **JC13**. Lè gen anpil koripsyon. | (1) Li ta gen rezon sifizan pou lapolis pran pouvwa a | (2) Li pa ta gen rezon sifizan pou lapolis pran pouvwa a | Pa konnen  **[PA LI REPONS LA]**  (888888) | Pa reponn  **[PA LI REPONS LA]**  (988888) | Pa aplike  **[PA LI REPONS LA]**  (999999) |

| **JC13COVID.** Lè gen yon ijans sante piblik tankou nan ka kowonaviris la | (1) Li ta jistifye pou lapolis pran pouvwa a | (2) Li pa ta jistifye pou lapolis pran pouvwa a | Pa konnen  **[PA LI]**  (888888) | Pa reponn **[PA LI]**  (988888) |
| --- | --- | --- | --- | --- |

| **KESYONE A** | | | | | |
| --- | --- | --- | --- | --- | --- |
| **JCCOV1.** Èske ou kwè ke lè gen yon ijans sante piblik tankou kowonaviris la, li jistifye pou prezidan peyi a ranvwaye eleksyon yo? | (1) Wi li jistifye | (2) Non, li pa jistifye | Pa konnen **[PA LI REPONS LA]**  (888888) | Pa reponn  **[PA LI REPONS LA]**  (988888) | Pa aplike  **[PA LI REPONS LA]**  (999999) |

| **KESYONE B** | | | | | |
| --- | --- | --- | --- | --- | --- |
| **JCCOV2.** Èske ou kwè ke lè gen anpil vyolans li jistifye pou prezidan peyi a ranvwaye eleksyon yo? | (1) Wi li jistifye | (2) Non li pa jistifye | Pa konnen  **[PA LI REPONS LA]**  (888888) | Pa reponn  **[PA LI REPONS LA]**  (988888) | Pa aplike  **[PA LI REPONS LA]**  (999999) |

| **VIC1EXT.** Konnye a, ann pale lòt bagay, èske w te viktim kèk zak kriminèl nan 12 mwa ki sot pase yo? Sa vle di, eske w te viktim yon vòl, kase kay, agresyon, fwod, chantaj, kraponnay, eskokri, anlèvman oswa kidnapin, menas vyolan oswa nenpòt lòt kalite krim nan 12 mwa ki sot pase yo?  (1) Wi (2) Non  (888888) Pa konnen **[PA LI REPONS LA]**  (988888) Pa reponn **[PA LI REPONS LA]** |  |
| --- | --- |

| **AOJ11**. Ann pale de kote oswa zòn wap viv la. Ann panse ki posiblite pou w ta viktim yon zak kriminèl, eske w santi w: toujou an sekirite, preske toujou an sekirite, pa toujou an sekirite, osinon pa janm an sekirite?  (1) Toujou an sekirite  (2) Preske toujou an sekirite  (3) Pa toujou an sekirite  (4) Pa janm an sekirite  (888888) Pa konnen **[PA LI REPONS LA]** (988888) Pa reponn **[PA LI REPONS LA]** |  |
| --- | --- |

| **IVOL24.** Eske gen gwoup gang kriminèl, gang nan zòn kote wap viv la?  (0) Non **[Ale nan B0]**  (1) Wi **[Kontinye]**  (888888) Pa konnen **[PA LI REPONS LA] [Ale nan B0]**   (988888)  Pa reponn **[PA LI REPONS LA] [Ale nan B0]** |
| --- |
| **GANG10.** Nan kèk zòn nan peyi a gang yo ap fè divès aktivite. Èske w ka di gang yo bay moun nan zòn bò lakay ou sekirite?  (1) Wi (2) Non  (888888) Pa konnen **[PA LI REPONS LA]**        (988888)  Pa reponn **[PA LI REPONS LA]**  (999999) Pa aplike **[PA LI REPONS LA]** |
| **GANG11.** Nan sa ki gen pou wè ak Ede moun yo jwenn bagay de baz tankou manje, Èske w ka di gang yo bay moun nan zòn bò lakay ou jan de sèvis sa? (1) Wi (2) Non  (888888) Pa konnen **[PA LI REPONS LA]**          (988888)  Pa reponn **[PA LI REPONS LA]**  (999999) **[PA LI REPONS LA]** Pa aplike |
| **GANG12.** Jwe wòl lidè kominotè. Èske w ka di gang yo jwe wòl lidè kominotè nan zòn bò lakay ou?  (1) Wi (2) Non  (888888) Pa konnen **[PA LI REPONS LA]**        (988888)  Pa reponn **[PA LI REPONS LA]**  (999999) **Pa aplike** **[PA LI REPONS LA]** |

| **B0T.** Mwen pral mande w pou reponn yon seri kesyon pandan wap chwazi yon nimewo nan yon nechèl ki komanse nan 1 ki rive nan 7. 1 se nimewo ki pi ba, li vle di ”PA DI TOU”. 7 se nimewo ki pi wo a, li vle di “ANPIL”. Men si opinyon w se antre «PA DI TOU» ak «AK ANPIL» ou ka chwazi yon nimewo ant 1 ak 7. Pou komanse, nan ki nivo ou renmen gade televizyon? Dim nimewo a. **[Asire w ke moun nan konprann byen kijan pou li reponn]**. | | | | | | | | | | |
| --- | --- | --- | --- | --- | --- | --- | --- | --- | --- | --- |
| 1 | 2 | 3 | 4 | | 5 | 6 | 7 | 888888 | 988888 | |
| **Pa di tou** | | | | **Anpil** | | | | Pa konnen  **[PA LI REPONS LA]** | Pa reponn **[PA LI REPONS LA]** | |
| **[Ekri nimewo 1-7, 888888=Pa konnen 988888=Pa reponn]** | | | | | | | | | | |
| Mwen pral poze w kèk kesyon. Mwen pral mande w pou w itilize nimewo yo bay nan nechèl la pou reponn. Sonje, ou ka itilize nenpòt nimewo.  **B1**. Nan ki nivo w kwè tribinal jistis an Ayiti bay yon bon jijman? **(Tyeke:** Si w kwè tribinal yo pa bay bon jijman di tou, chwazi nimewo 1; si ou kwè yo bay anpil bon jijman, chwazi nimewo 7 oswa yon nimewo ant 1 ak 7.) | | | | | | | | | |  |
| **B2**. Nan ki nivo ou genyen respè pou enstitisyon politik peyi d Ayiti? | | | | | | | | | |  |
| **B3**. Nan ki nivo ou kwè dwa fondamantal sitwayen yo byen pwoteje nan sistèm politik peyi d Ayiti? | | | | | | | | | |  |
| **B4**. Nan ki nivo ou fyè pou viv anba systèm politik gouvèvnman peyi d Ayiti? | | | | | | | | | |  |
| **B6**. Nan ki nivo ou panse ke moun dwe sipòte systèm politik gouvèvnman peyi d Ayiti? | | | | | | | | | |  |
| **BESS1.** Nan ki nivo ou panse ke lòt moun ta ka pwofite de ou si yo gen posibilite pou fè sa? | | | | | | | | | |  |

| **M1.** Ann pale de gouvènman ki sou pouvwa a, eske w ta di travay Jovenel Moise ap fè a: **[Li repons yo]**  (1) Li bon anpil (2) Li bon (3) Li pa pi mal (4) Li mal (5) Li pi mal  (888888) Pa konnen **[PA LI REPONS LA]** (988888) Pa reponn **[PA LI REPONS LA]** |  |
| --- | --- |

| **HAIM6.** Pwosesis elektoral an Ayiti, ou ka di, li bon anpil, li bon, li pa pi mal, li mal, li pi mal?  (1) Li bon anpil (2) Li bon (3) Li pa pi mal (4) Li mal (5) Li pi mal  (888888) Pa konnen **[PA LI REPONS LA]**  (988888) Pa reponn **[PA LI REPONS LA]** |  |
| --- | --- |

| Nan kesyon kap vini a map mande ankò pou w ban mwen yon repons ak yon nimewo. Nou pral itilize yon nechèl ki kòmanse nan nimewo 1 ki vle di “Pa dakò di tou” epi ki rive nan nimewo 7 ki vle di ou “ Dakò nèt”. Yon nimewo ki ant 1 ak 7 vle di ke w on ti jan pa dakò oswa w pa tèlman dakò. | | | | | | | | |
| --- | --- | --- | --- | --- | --- | --- | --- | --- |
| 1 | 2 | 3 | 4 | 5 | 6 | 7 | 888888 | 988888 |
| **Pa dakò di tou Dakò nèt** | | | | | | | **Pa konnen**  **[PA LI REPONS LA]** | **Pa reponn**  **[PA LI REPONS LA]** |

**[Ekri nimewo 1-7, 888888=Pa konnen 988888=Pa reponn]**

| **ING4.** Demokrasi kapab genyen pwoblèm men li pi bon pase nenpòt kèk lòt fòm gouvènman. Nan ki nivo ou dakò osinon pa dakò ak pawòl sa yo? |  |
| --- | --- |

| **KESYONE A** | |
| --- | --- |
| **EXC7.** Daprè eksperyans pa w oswa sa ou konn tande, eske w ta di koripsyon kay fonksyonè leta/piblik yo…? **[Li repons yo]**  (1) Komen anpil (2) komen (3) Pa komen (4) Pa komen di tou  (888888) Pa Konnen **[PA LI REPONS LA]**  (988888) Pa reponn **[PA LI REPONS LA]**  (999999) Pa aplike **[PA LI REPONS LA]** |  |

| **KESYONE B** | |
| --- | --- |
| **EXC7NEW.** An panse sou politisyen ayisyen. Konbyen nan yo ou panse ki nan zak koripsyon **[Li repons yo]**  (1) Okenn nan yo  (2) Pi piti ke mwatye nan yo  (3) Mwatye nan yo  (4) Plis ke mwatye nan yo  (5) Yo tout  (888888) **[PA LI REPONS LA]** Pa Konnen  (988888) **[PA LI REPONS LA]** Pa reponn  (999999) **[PA LI REPONS LA]** Pa aplike |  |

| **ESSH1.** Kouman sistyasyon sante ou ye an jeneral? Èske ou ta di...**[Li repons yo]**  (1) Li bon anpil (2) Li bon (3) Li pa pi mal (4) Li mal (5) Li mal anpil  (888888) Pa konnen **[PA LI REPONS LA]** (988888) Pa reponn **[PA LI REPONS LA]** |  |
| --- | --- |

| **HC3.** Pandan 12 mwa ki sot pase yo, depi Avril/Me 2019 jiska jodia, èske ou menm oswa kek moun kap viv nan kay sa te bezwen swen medikal?  (1) Wi **[Kontinye]** (2) Non **[Ale nan SD6NEW2H ]**  (888888) **[PA LI REPONS LA ]** Pa Konnen **[Ale nan SD6NEW2H]**  (988888) **[PA LI REPONS LA ]** Pa reponn **[Ale nan SD6NEW2H]** |  |
| --- | --- |
| **HC4.** Dènye fwa ou menm, oswa yon moun kap viv nan kay la te bezwen swen medikal, ki moun ou te al wè?   1. Doktè general **[Kontynye]** 2. Yon doktè espesyalist **[Kontynye]** 3. Yon enfimyè **[Kontynye]** 4. Yon famasyen **[Kontynye]** 5. Yon doctè Fèy **[Kontynye]** 6. Boko / mambo **[Kontynye]** 7. Fanmi/ Vwazen **[Kontynye]** 8. Lòt moun **[Kontynye]** 9. M pat we pyes moun **[Ale nan SD6NEW2H]**   (888888) **[PA LI REPONS LA]** Pa Konnen **[Ale nan SD6NEW2H]**  (988888) **[PA LI REPONS LA]** Pa reponn **[Ale nan SD6NEW2H]**  (999999) **[PA LI REPONS LA]** Pa aplike |  |

| **HC5.** Ki kote ou te resevwa sèvis medikal la? **[Pa li Repons yo]**  (1) Lopital Prive **[Kontynye]**  (2) Lopital Piblik **[Kontynye]**  (3) Yon Klinic Kominotè **[Kontynye]**  (4) Yon sant sante Piblik **[Kontynye]**  (5) Klinik PRive **[Kontynye]**  (6) Klinik yon ONG **[Kontynye]**  (7) Nan biwo moun mwen te konsilte a **[Ale nan SD6NEW2H]**  (8) Famasy **[Ale nan SD6NEW2H]**  (9) Lakay mwen **[Ale nan SD6NEW2H]**  (10) Lòt kote **[Ale nan SD6NEW2H]**  (888888) **[PA LI REPONS LA]** Pa Konnen  (988888) **[PA LI REPONS LA]** Pa reponn  (999999) **[PA LI REPONS LA]** Pa aplike |  |
| --- | --- |
| **HC7.** Nan ki nivo ou te satisfè ak sèvis sante sa ou te resevwa a?  (1) Satisfè anpil (2) On tijan Satisfè  (3) On tijan pa satisfè (4) Pa satisfè ditou  (888888) **[PA LI REPONS LA]** Pa Konnen  (988888) **[PA LI REPONS LA]** Pa reponn  (999999) **[PA LI REPONS LA]** Pa aplike |  |

| **SD6NEW2H.** Lè wap panse nan kalite sèvis medikal ak sante piblik yo? Èske w ta di ou … **[Li repons yo]**  (1) Satisfè nèt (2) Satisfè  (3) Pa satisfè (4) Pa satisfè di tou  (888888) Pa konnen **[PA LI REPONS LA]**  (988888) Pa reponn **[PA LI REPONS LA]**  (999999) Pa aplike (Pa itilize sèvis la) **[PA LI REPONS LA]** |  |
| --- | --- |

| **KESYONE A** |  |
| --- | --- |
| **POL1A.** Nan ki nivo ou enterese nan politik: anpil, on ti jan, pa anpil oswa pa di tou?  (1) Anpil (2) On ti jan (3) Pa anpil (4) Pa di tou  (888888) Pa Konnen **[PA LI REPONS LA]** (988888) Pa reponn **[PA LI REPONS LA]**  (999999) **[PA LI REPONS LA]** Pa aplike |  |
| **VB20A.** Si pwochèn eleksyon prezidansyèl yo ta nan semèn sa a, ki sa ou tap fè? **[Li repons yo]**  (1) Ou pa tap vote  (2) Ou tap vote pou kandida oubyen pati ki sou pouvwa kounya  (3) Ou tap vote pou kandida oubyen pati ki pa sou pouvwa kounya  (4) Ou tap vote men ou tap vote blan ou byen ou ta anile vòt ou a  (888888) Pa Konnen **[PA LI REPONS LA]**  (988888) Pa reponn **[PA LI REPONS LA]**  (999999) **[PA LI REPONS LA]** Pa aplike |  |

| **KESYONE B** |  |
| --- | --- |
| **VB20B.** Si pwochèn eleksyon prezidansyèl yo ta nan semèn sa a, ki sa ou tap fè? **[Li repons yo]**  (1) Ou pa tap vote  (2) Ou tap vote pou kandida oubyen pati ki sou pouvwa kounya  (3) Ou tap vote pou kandida oubyen pati ki pa sou pouvwa kounya  (4) Ou tap vote men ou tap vote blan ou byen ou ta anile vòt ou a  (888888) Pa Konnen **[PA LI REPONS LA]**  (988888) Pa reponn **[PA LI REPONS LA]**  (999999) **[PA LI REPONS LA]** Pa aplike |  |
| **POL1B.** Nan ki nivo ou enterese nan politik: anpil, on ti jan, pa anpil oswa pa di tou?  (1) Anpil (2) On ti jan (3) Pa anpil (4) Pa di tou  (888888) Pa Konnen **[PA LI REPONS LA]** (988888) Pa reponn **[PA LI REPONS LA]**  (999999) **[PA LI REPONS LA]** Pa aplike |  |

| **KESYONE A** | | | | | | |
| --- | --- | --- | --- | --- | --- | --- |
| Ann chanje sijè… | Plis | Menm kantite | Pi piti | Pa Konnen **[PA LI REPONS LA ]** | Pa reponn **[PA LI REPONS LA ]** | Pa aplike **[PA LI REPONS LA]** |
| **HAIRIG1.** Èske ou panse ke ou konnen plis bagay, mennm kantite bagay osinon pi piti bagay sou dwa ou genyen kòm moun nan 12 mwa ki sòt pase yo? | 1 | 2 | 3 | 888888 | 988888 | 999999 |

| **KESYONE B** | | | | | | |
| --- | --- | --- | --- | --- | --- | --- |
| Ann chanje sijè… | Plis | Menm kantite | Pi piti | Pa Konnen **[PA LI REPONS LA ]** | Pa reponn **[PA LI REPONS LA ]** | Pa aplike **[PA LI REPONS LA]** |
| **HAIRIG5.** Èske ou santi ou konnen plis, menm kantite ou pi piti bagay sou dwa ou genyen pou w jwenn jistis nan 12 mwa ki sot pase yo? | 1 | 2 | 3 | 888888 | 988888 | 999999 |

| **WF1.** Eske ou menm oswa Kèk lòt moun kap viv nan kay sa resevwa regilyèman èd tankou lajan, manje osinon lòt pwodui nan men gouvènman a, ki difèran de pansion osinon sekirite sosyal?  (1) Wi (2) Non  (888888) Pa konnen [**PA LI REPONS LA]** (988888) Pa reponn [**PA LI REPONS LA]** |  |
| --- | --- |

| **KESYONE B**  Kounye a mwen ta renmen mande ou kèk kesyon ki gen pou wè ak sityasyon kowonaviris la. | |
| --- | --- |
| **COVID1B.** Nan ki nivo ou panse ke epidemi kowonaviris la se yon pwoblèm ki grav pou Ayiti? **[Li repons yo]**  (1) Li grav anpil (2) Ontijan grav (3) Li pa si grav (4) Li pa grav ditou  (5) M pa panse anpil ak sa  (888888) **[PA LI REPONS LA]** Pa konnen  (988888) **[PA LI REPONS LA]** Pa reponn  (999999) **[PA LI REPONS LA]** Pa aplike |  |

| **KESYONE B** | | | | | | | |
| --- | --- | --- | --- | --- | --- | --- | --- |
|  | **Preokipe anpil** | **Ontijan preokipe** | **On ti kras preokipe** | **Pa preokipe ditou** | **[PA LI REPONS LA]**  **Pa konnen** | **[PA LI REPONS LA]**  **Pa reponn** | **[PA LI REPONS LA]**  **Pa aplike** |
| **COVID2AB.** Nan ki nivo ou preokipe ke ou menm oswa yon moun kap viv nan kay sa a ta ka vin malad ak kowonaviris? **[Li repons yo]** | 1 | 2 | 3 | 4 | 888888 | 988888 | 999999 |
| **COVID2BB.** Nan ki nivo ou preokipe ke sityasyon ekonomik moun kap viv nan kay sa a kapab afekte negativman pa kowonaviris la? **[Li repons yo]** | 1 | 2 | 3 | 4 | 888888 | 988888 | 999999 |
| **COVID2CB.** Nan ki nivo ou preokipe ke moun kap viv nan kay sa a kapab gen difikilte poul jwenn bagay bazik tankou manje, medikaman akoz kowonaviris la? **[Li repons yo]** | 1 | 2 | 3 | 4 | 888888 | 988888 | 999999 |

| **KESYONE B** | |
| --- | --- |
| **COVID3B.** Kouman ou ta evalye pèfòmans gouvènman santral la nan fason lap fè fas ak epidemi kowonaviris la?  (1) Li bon anpil (2) Li bon (3) Pa pi mal (4) Li mal (5) Li mal anpil  (888888) **[PA LI REPONS LA] Pa konnen** (988888) **[PA LI REPONS LA]** Pa reponn  (999999) **[PA LI REPONS LA]** Pa aplike |  |
| **COVID4B.** Kowonaviris la ap layite kol an Ayiti, kiyès ou panse **ki gen plis responsabilite** pou kantite ka kap ogmante yo? **[PA LI LIST REPONS SA YO. EKRI SÈLMAN YON SEL REPONS]**  (1) Gouvènman sa a  (2) Nou menm, pèp ayisyen  (3) Moun ki gen lajan, boujwa peyi a  (4) Etazini  (5) La chinn  (6) Okenn moun pa gen tò nan sa  (7) Se bondye/se volontel  (77) Lòt repons  (888888) **[PA LI REPONS LA]** Pa konnen  (988888) **[PA LI REPONS LA]** Pa reponn  (999999) **[PA LI REPONS LA]** Pa aplike |  |

| **KESYONE B**  Èske ou ka dim si ou te pran youn nan desizyon sayo akoz de epidemi kowonaviris la? **[Randomize items]** | |
| --- | --- |
| **COVID6B.** Eske ou te anile plan pou patisipe nan reyinyon ki ta pral gen anpil moun tankou evènman sosyal, al legliz?  (1) Wi (2) Non  (888888) **[PA LI REPONS LA]** Pa konnen (988888) **[PA LI REPONS LA]** Pa reponn  (999999) **[PA LI REPONS LA]** Pa aplike |  |
| **COVID7B.** Èske w te kenbe pi gwo distans ant ou menm ak lòt moun lè w sòti nan lari?  (1) Wi (2) Non  (888888) **[PA LI REPONS LA]** Pa konnen (988888) **[PA LI REPONS LA]** Pa reponn  (999999) **[PA LI REPONS LA]** Pa aplike |  |
| **COVID8B.** Èske w lave men ou pi souvan avèk dlo, savon oswa dezenfektan?  (1) Wi (2) Non  (888888) **[PA LI REPONS LA]** Pa konnen (988888) **[PA LI REPONS LA]** Pa reponn  (999999) **[PA LI REPONS LA]** Pa aplike |  |

| **KESYONE B** | |
| --- | --- |
| **COVID14B.** Lè ou konsidere moun ki gen kowonaviris la, èske ou ta di ke yo merite maladi a akoz de jan yo menen vi yo, oswa yo pa gen oken responsabilite nan sa?  (1) Wi yo merite li (2) Non yo pa gen okenn responsabilite nan maladi a  (888888) **[PA LI REPONS LA]** Pa konnen (988888) **[PA LI REPONS LA]** Pa reponn  (999999) **[PA LI REPONS LA]** Pa aplike |  |

| **ED.** Ki dènye klas ou te gen chans fè lekòl?  _______ Ane _________________ (Primè**,** Segondè, Inivèsitè**) _____________** Total Ane  **[Itilize tab ki parèt anba pou ka jwenn k**ò**d la]** |
| --- |

| (0) 0 lane | Ankenn nivo |
| --- | --- |
| (1) 1 lane | Preskolè |
| (2) 2lane | Preparatwa 1 / 1 A.F. |
| (3) 3 lane | Preparatwa 2 / 2 A.F. |
| (4) 4 lane | Elemantè 1 / 3 A.F. |
| (5) 5 lane | Elemantè 2 / 4 A.F. |
| (6) 6 lane | Mwayen 1 / 5 A.F. |
| (7) 7 lane | Mwayen 2 / 6 A.F. |
| (8) 8 lane | Sizyèm / 7 A.F. |
| (9) 9 lane | Senkyèm / 8 A.F |
| (10) 10 lane | Katryèm / 9 A.F. |
| (11) 11 lane | Twazyèm |
| (12) 12 lane | Segonn |
| (13) 13 lane | Reto |
| (14) 14 lane | Filo |
| (15) 15 lane | Inivèsite 1 |
| (16) 16 lane | Inivèsite 2 |
| (17) 17 lane | Inivèsite 3 |
| (18) 18 lane | Plis ke 4 lan**e** |
| (888888) | **[PA LI REPONS LA]** Pa konnen |
| (988888) | **[PA LI REPONS LA]** Pa reponn |

| **Q5B**. Ki enpòtans relijyon genyen nan lavi w? **[Li repons yo]**  (1) Trè enpòtan anpil (2) On ti jan enpòtan (3) On ti jan pa enpòtan  (4) Pa enpòtan di tou  (888888) **[PA LI REPONS LA]**Pa konnen  (988888) **[PA LI REPONS LA]**Pa reponn |  |
| --- | --- |
| **OCUP4A.** Aktyèlman, kisa wap fè? **[Li repons yo]**  (1) Wap travay?  (2) Ou nan konje men ou gen travay?  (3) W ap chèche travay tankou mèt janjak?  (4) W ap etidye?  (5) W ap okipe kay la sèlman?  (6) Ou se retrete, pansyonè, enkapasite pèmanan (pa nan eta pou li travay)?  (7) Ou pap travay epi ou pap chèche travay?  (888888) Pa konnen **[PA LI REPONS LA]** **[**  (988888) Pa reponn **[PA LI REPONS LA]** |  |
| **Q10A.** Eske ou menm osinon yon lòt moun ki ap viv nan kay sa resevwa transfè lajan oswa sipò ekonomik ki sòti nan peyi letranje?  (1) Wi (2) Non  (888888) **[PA LI REPONS LA]**Pa konnen  (988888) **[PA LI REPONS LA]** Pa reponn |  |

| **Q14.** Eske w genyen lide ale viv osinon travay nan peyi letranje nan twa lane kap vini yo?  (1) Wi (2) Non  (888888) **[PA LI REPONS LA]** Pa konnen  (988888) **[PA LI REPONS LA]** Pa reponn |  |
| --- | --- |
| **Q10E.** Nan 2 lane ki fèk sot pase yo, èske moun lakay ou fè: **[Li repons yo]**  (1) Plis kòb?  (2) Menm valè kòb?  (3) Mwens kòb?  (888888) **[PA LI REPONS LA]** Pa konnen  (988888) **[PA LI REPONS LA]** Pa reponn |  |

| Konnye a, mwen pral li kèk kesyon sou koze manje nan kay la. | | | | | |
| --- | --- | --- | --- | --- | --- |
|  | **Non** | **Wi** | Pa konnen **[PA LI REPONS LA]** | Pa reponn **[PA LI REPONS LA]** |  |
| **FS2.** Nan twa mwa ki fèk sot pase yo, poutèt pa gen lajan oswa lòt resous, èske pafwa moun lakay ou pat gen manje? | 0  **[Ale nan GI0N]** | 1  **[Kontinye]** | 888888  **[Ale nan GI0N]** | 988888  **[Ale nan to GI0N]** |  |
| **FS2COVID.** Sa te rive akòz kowonaviris la oswa se lòt rezon ki eksplike sa?   1. Kowonaviris 2. Lòt rezon   (888888) **[PA LI REPONS LA]** Pa konnen  (988888) **[PA LI REPONS LA]** Pa reponn  (999999) **[PA LI REPONS LA]** Pa aplike | | | | |  |

| **GI0N.** Chak kilè w tande nouvèl nan radyo, gade nouvèl nan televizyon, oswa li nouvèl nan jounal oswa sou entenèt? **[Li repons yo]:**  (1) Chak jou (2) Kèk fwa pa semèn (3) Kèk fwa pa mwa  (4) Kèk fwa pa ane (5) Pa janm fè sa  (888888) **[PA LI REPONS LA]** Pa konnen  (988888) **[PA LI REPONS LA]** Pa reponn |  |
| --- | --- |

Èske ou ka dim si ou gen bagay sa yo la kay ou: **[Li tout repons yo]**

| **R3**. Frijidè (Refrijeratè) | (0) Non | | | | (1) Wi | | Pa konnen **[PA LI REPONS LA]**  (888888) | | Pa reponn **[PA LI REPONS LA]**  (988888) | |
| --- | --- | --- | --- | --- | --- | --- | --- | --- | --- | --- |
| **R4.** Telefòn fiks | (0) Non | | | | (1) Wi | | Pa konnen **[PA LI REPONS LA]**  (888888) | | Pa reponn **[PA LI REPONS LA]**  (988888) | |
| **R4A**. Telefòn potab, selilè (Telefòn entèlijan ladan tou) | (0) Non | | | | (1) Wi | | Pa konnen **[PA LI REPONS LA]**  (888888) | | Pa reponn **[PA LI REPONS LA]**  (988888) | |
| **R5**. Machin oswa kamyon **[Si moun nan pa di konbyen make yon (1)]** | (0) Non | (1) Yon | (2) De | | (3) wa oswa plis pase twa | | Pa konnen **[PA LI REPONS LA]**  (888888) | | Pa reponn **[PA LI REPONS LA]**  (988888) | |
| **R6**. Machin pou lave rad | (0) Non | | | | (1) Wi | | Pa konnen **[PA LI REPONS LA]**  (888888) | | Pa reponn **[PA LI REPONS LA]**  (988888) | |
| **R7**. Fou micro-onde/Maykowev | (0) Non | | | | (1) Wi | | Pa konnen **[PA LI REPONS LA]**  (888888) | | Pa reponn **[PA LI REPONS LA]**  (988888) | |
| **R8**. Motosiklèt | (0) Non | | | | (1) Wi | | Pa konnen **[PA LI REPONS LA]**  (888888) | | Pa reponn **[PA LI REPONS LA]**  (988888) | |
| **R12**. Dlo potab (tiyo) anndan kay la | (0) Non | | | | (1) Wi | | Pa konnen **[PA LI REPONS LA]**  (888888) | | Pa reponn **[PA LI REPONS LA]**  (988888) | |
| **R14**. Douch anndan kay la | (0) Non | | | | (1) Wi | | Pa konnen **[PA LI REPONS LA]**  (888888) | | Pa reponn **[PA LI REPONS LA]**  (988888) | |
| **R15.** Òdinatè (Asepte tablet tou) | (0) Non | | | | (1) Wi | | Pa konnen **[PA LI REPONS LA]**  (888888) | | Pa reponn **[PA LI REPONS LA]**  (988888) | |
| **R18.** Entenèt nan kay la (Sa enkli entenet nan telefòn li osinon nan Tablet) | (0) Non | | | | (1) Wi | | Pa konnen **[PA LI REPONS LA]**  (888888) | | Pa reponn **[PA LI REPONS LA]**  (988888) | |
| **R1**. Televizyon | (0) Non **[Ale nan PSC1]** | | | | (1) Wi **[Kontinye]** | | Pa konnen **[PA LI REPONS LA]**  (888888) | | Pa reponn **[PA LI REPONS LA]**  (988888) | |
| **R16.** Televizyon plat oswa flat. | (0) Non | | | (1) Wi | | Pa konnen **[PA LI REPONS LA]**  (888888) | | Pa reponn **[PA LI REPONS LA]**  (988888) | | Pa aplike **[PA LI REPONS LA]**  **(999999)** |

| **PSC1.** Ki kote pi fò dlo moun lakay ou bwè sòti? **[PA LI REPONS YO.. Make on sel repons] [Si moun nan di dlo soti plizye kote, mandel pou li di w sa li plis itilize] [si moun nan di dlo a s**ò**ti nan tiyo, mande li si se tiyo 1) ki rive andan kay la 2) osinon dey**ò kay la**]**  (01) Tiyo ki rive jouk nan kay la  (02) Tiyo nan lakou a  (03) Yon koneksyon nou fè sou tiyo piblik (konbelan)  (04) Fontèn piblik  (05) Pwi (ki itilize pomp)  (06) Yon sous ki bare (ki pa itilize pomp)  (07) Yon sous ki pa bare (ki pa itilize pomp)  (08) Dlo kap koule ki pwotege  (09) Dlo kap koule ki pa pwotege  (10) Dlo lapli nou gade nan siten  (11) Achte boutèy dlo  (12) Achte Bokit dlo  (13) Achte Kamyon dlo  (14) Rivyè, canal,  (77) Lòt  (888888) **[PA LI REPONS LA ]** Pa konnen  (988888) **[PA LI REPONS LA ]** Pa reponn |  |
| --- | --- |
| **PSC2.** Ki bò pi fò dlo yo itilize pou fè lòt bagay lakay ou soti (tankou pou fe mange, lave men)? **[PA LI repons yo. Make Sèlman yon repons.] [Si moun nan di plis pase yon sel kote, mande pou li diw sa li itilize pli souvan an]**  **[Si moun nan di tiyo mande li si (1) se anndan kay la (2) osinon dey**ò kay la**]**  (01) Tiyo ki rive jouk nan kay la **[Kontinye]**  (02) Tiyo nan lakou a **[Kontinye]**  (03) Yon koneksyon nou fè sou tiyo piblik (konbelan) **[Kontinye]**  (04) Fontèn piblik **[Ale nan PSC11]**  (05) Pwi (ki itilize pomp)**[Ale nan PSC11]**  (06) Yon sous ki bare (ki pa itilize pomp) **[Ale nan PSC11]**  (07) Yon sous ki pa bare (ki pa itilize pomp) **[Ale nan PSC11]**  (08) Dlo kap koule ki pwotege **[Ale nan PSC11]**  (09) Dlo kap koule ki pa pwotege **[Ale nan PSC11]**  (10) Dlo lapli nou gade nan sitèn **[Ale nan PSC11]**  (11) Achte Boutèy dlo **[Ale nan PSC11]**  (12) Achte boutèy dlo **[Ale nan PSC11]**  (13) Achte Kamyon dlo **[Ale nan PSC11]**  (14) Rivyè, canal, **[Ale nan PSC11]**  (77) Lòt **[Ale nan PSC11]**  (888888) **[PA LI REPONS YO]** Pa konnen **[Ale nan PSC11]**  (988888) **[PA LI REPONS YO] Pa reponn [Ale nan PSC11]** |  |
| **[Mande yo kesyon sa sèlman si gen tiyo nan kay yo– si repons yo te bay nan PSC2 te (01) (02) oswa (03)]**  **PSC7.** Konbyen jou pa semèn ou resevwa dlo nan tiyo?  (0) Mwens ke on fwa pa semèn  (1) Yon jou pa semèn  (2) De jou pa semèn  (3) Twa jou pa semèn  (4) Kat jou pa semèn  (5) Sink jou pa semèn  (6) Sis jou pa semèn  (7) Sèt jou pa semèn  (888888) **[PA LI REPONS YO]** Pa konnen  (988888) **[PA LI REPONS YO]** Pa reponn  (999999) **[PA LI REPONS YO]** Pa aplike |  |
| **[Mande yo kesyon sa** èè**lman si gen tiyo nan kay yo– si repons yo te bay nan PSC2 te (01) (02) oswa (03)]**  **PSC8. Konbyen** è**d tan pa jou dlo a rive nan jou tiyo a bay dlo**?  Ekri kantite èd tan____________________________ **[Atansyon, si li reponn nan minit oswa yon pati nan l**è **a fè repons lan rive nan le ki pi pwoch la]**  **[Nou ap pale de tiyo , san konte resevwa dlo ki ka gen nan kay la]**  **[Kantite** è**dtan maxim**ò**m pou asepte : 24]**  (888888) **[PA LI REPONS YO]** Pa konnen  (988888) **[PA LI REPONS YO]** Pa reponn  (999999) **[PA LI REPONS YO]** Pa aplike |  |
| **[Mande yo kesyon sa sèlman si gen tiyo nan kay yo– si repons yo te bay nan PSC2 te (01) (02) oswa (03)]**  **PSC9.** Nan kat semèn ki sot pase yo, konbyen fwa sèvis dlo nan kay la pat fonksyone? **[Ekri konbyen fwa]** __________________  **[Kantite maksimom pou w asepte: 50]**  (888888) **[PA LI REPONS YO]** Pa konnen  (988888) **[PA LI REPONS YO]** Pa reponn  (999999) **[PA LI REPONS YO]** Pa aplike |  |
| **PSC11.** Twalèt nan kay la konekte … **[Li repons yo]**  (1) Ak twou egou **[Ale nan PSC12]**  (7) Ak on sistèm kote yo trete li **[Ale nan Skip to PSC12]**  (2) Nan twou septik ki deyo kay la **[Ale nan PSC12]**  (3) Ak yon tib ki mennen nan yon ravin dlo **[Ale nan PSC12]**  (4) Lòt [Li konekte epi lale lòt kote] **[Ale nan PSC12]**  (5) Twalèt la ale on kote moun nan pa konnen **[Ale nan PSC12]**  (6) Yon twou ki sèvi kòm latrin **[Kontinye]**  (888888) **[PA LI REPONS LA]** Pa konnen **[Ale nan PSC12]**  (988888) **[PA LI REPONS LA]** Pa reponn **[Ale nan PSC12]** |  |
| **[Mande kesyon sa Sèlman si twalèt la pa konekte ak oken system, repons (6) nan PSC11]**  **PSC11A.** Kisa ou itilize lakay ou pou ale nan twalèt? **[Li repons yo]**  (1) Latrin ki ouvri  (2) Twou latrin ki gen kote pou chita  (3) Twou latrin ki pa gen kote pou ou chita  (4) Twalèt kote yap fe Konpos  (5) Bokit  (6) Latrin pandye  (7) Nou ale nan rage, pa gen twalèt  (77) Lòt twalè  (888888) **[PA LI REPONS LA]** Pa konnen  (988888) **[PA LI REPONS LA]** Pa reponn  (999999) **[PA LI REPONS LA]** Pa aplike |  |
| **PSC12.** Eske gen plizyè kay ki itilize twalèt sa?  (1) Wi (2) Non  (888888) **[PA LI REPONS LA]** Pa konnen  (988888) **[PA LI REPONS LA]** Pa reponn |  |
| **PSC13.** Souple, èske ou ka di mwen ki sa nou fè pou debarase nou de fatra ki fèt nan kay la?  **[PA LI repons yo. Make tout repons ki aplikab] [Eseye pou wè si gen sèvis ramasaj fatra de fason f**ò**mel (01) osinon enf**ò**mèl (02), oswa si yo resikle de fason fomèl (12) osinon enf**ò**mel (13)]**  (01) Sèvis ramasaj fatra (s**è**vis fòm**è**l)  (02) Gen moun ki pran fatra a (sevis enfòm**è**l)  (03) Genyen yon kontene nan zon nan kote nou jete fatra  (04) No jete nan yon depotwa minisipal  (05) Antere li  (06) Nou fè konpost avèk li  (07) Nou boule li  (08) Nou jetel nan teren vid, nan canal, rivye  (09) Nou jetel nempot lòt kote  (10) Nou resikle nan kay la (se pa konpost)  (11) Nou potel on kote kote yo resikle li  (12) La meri fè ramasaj fatra pou resikle (de fason fòm**è**l)  (13) Moun fè Ramasaj fatra pou resikle (de fason enfòm**è**l)  (888888) **[PA LI REPONS LA]** Pa konnen  (988888) **[PA LI REPONS LA]** Pa reponn |  |
| **PSC3.** M pral mansyone Kèk pwoblem ke anpil nan nou Ayisyen te afronte nan ane ki sòt pase yo. Kiyès nan yo ou menm pèsonèlman oswa Kèk moun lakay ou te genyen nan twa lane ki sot pase yo? **[Li repons yo epi make tout sa ki aplikab]**  (0) **[PA LI REPONS LA]** Ankenn  (1) Sechrès ki te gen kom konsekans rate, dlo te koupe  (2) Elektrisite/koupan tap plede koupe  (3) Inondasyon  (888888) **[PA LI REPONS LA]** Pa konnen  (988888) **[PA LI REPONS LA]** Pa reponn |  |
| **PSC4.** Kiy**è**s ou panse ki gen pi gwo responsabilite a koz de sechrès sa a ki te menen nan rate dlo oswa koupi dlo? **[Li repons yo]**  (1) Gouvènman santral la  (3) Gouvènman komin nan (la meri)  (4) Konpayi dlo a /DINEPA  (5) Chanjman klimatik  (6) Nou menm  (77) Lòt  (888888) **[PA LI REPONS LA]** Pa konnen  (988888) **[PA LI REPONS LA]** Pa reponn |  |
| **PSC5.** Kiyès ou panse ki gen pi gwo responsabilite nan zafè pa gen kouran elektrik lan? **[Li repons yo]**  (1) Gouvènman santral la  (3) Gouvènman komin nan (la meri)  (4) Konpayi elektrisite a (EDH)  (5) Chanjman klimatik  (6) Nou menm  (77) Lòt  (888888) **[PA LI REPONS LA]** Pa konnen  (988888) **[PA LI REPONS LA]** Pa reponn |  |
| **PSC6.** Kiyès ou panse ki gen plis responsabilite pou inondasyon yo? **[Li repons yo]**  (1) Gouvènman santral la  (3) Gouvènman komin nan (la meri)  (5) Chanjman klimatik  (6) Nou menm  (77) Lòt  (888888) **[PA LI REPONS LA]** Pa konnen  (988888) **[PA LI REPONS LA]** Pa reponn |  |

| **LINEA1.** Se pou nimewo telefòn sa ye?  (1) Wi (2) Non  (888888) **[PA LI REPONS LA]** Pa konnen  (988888) **[PA LI REPONS LA]** Pa reponn |  |
| --- | --- |
| **LINEA2.** Se yon sèl moun ki iitilize liy nimewo telefòn sa-a, oswa se plizyè moun ki itilize li?  (1) Yon sèl moun itilize nimewo telefòn nan  (2) Plizyè moun itilize nimewo telefòn nan  (888888) **[PA LI REPONS LA]** Pa konnen  (988888) **[PA LI REPONS LA]** Pa reponn |  |
| **LINEA3.** Konbyen nimewo telefòn pòtab ou genyen? Dim sèlman konbyen nimewo telefòn pòtab ou genyen sou non ou _____________  (888888) **[PA LI REPONS LA]** Pa konnen  (988888) **[PA LI REPONS LA]** Pa reponn |  |

***Se tout Kèksyon mwen te genyen. Mèsi anpil pou kolaborasyon w.***

| **[Lè ou fini ankèt la, san ou pa mande moun lan anyen, reponn kesyon sa yo]** |
| --- |
| **NOISE1T.** Eske te gen lòt moun prezan pandan ou tap fè anket la, anplis de moun ou tap fè anket la avèk li a?  (0) Non  (1) Wi, li te lwen. Li pat afekte apèl la  (3) Wi, li te entèveni de tanzanta  (4) Wi, li te entèveni anpil, li te mete an danje antrevi a |
| **CONOCIM.** Itilize nechel sa. Di daprè ou nivo konesans politik moun ou sot fè ankèt la avel la.  (1) Nivol ro anpil (2) Nivol ro (3) Nivol mwayen  (4) Nivol ba (5) Nivol ba anpil |

| **Lè antrevia a fini:** _______ : ______ |  |
| --- | --- |
| **TI.** Konbyen tan ankèt la te dire ***[minit, gade nan paj # 1]*** _____________ |  |
| **INTID.** **Nimewo Identifikasyonl anketè a**: ____________ |  |
| **SEXI. Ekri seks ou:** (1) Gason (2) Fanm |  |

| *Mwen deklare ke ankèt sa sa te fèt ak moun mwen di a.* |
| --- |
| *Siyati anketè a __________________ Dat ____ /_____ /_____*  *Siyati sipèvisè a _______________________________________* |
| *Komantè: ________________________________________________________________________ _________________________________________________________________________________* |
| *[Pa pou PDA/Android] Siyati moun ki antre done yo _______________________* |
| *[Pa pou PDA/Android] Siyati moun ki te verifye done yo _______________________* |
